# Global transcriptome analysis and identification of genes involved in nutrients accumulation during seed development of rice tartary buckwheat (*Fagopyrum Tararicum*)

**DOI:** 10.1038/s41598-017-11929-z

**Published:** 2017-09-18

**Authors:** Juan Huang, Jiao Deng, Taoxiong Shi, Qijiao Chen, Chenggang Liang, Ziye Meng, Liwei Zhu, Yan Wang, Fengli Zhao, Shizhou Yu, Qingfu Chen

**Affiliations:** 1 Research Center of Guizhou Buckwheat Engineering and Technology, Research Center of Buckwheat Industry Technology, Guizhou Normal University, Baoshan Beilu 116, Guiyang, 550001 Guizhou P.R. China; 2 Agricultural Genomics Institute, Chinese Academy of Agricultural Sciences, Pengfei Road No. 7, Dapeng New District, Shenzhen, 518120 Guangdong, P.R. China; 3 Guizhou Academy of Tobacco Science, Longbatan Road 29, Guanshanhu District, Guiyang, 550081 Guizhou P.R. China

## Abstract

Tartary buckwheat seeds are rich in various nutrients, such as storage proteins, starch, and flavonoids. To get a good knowledge of the transcriptome dynamics and gene regulatory mechanism during the process of seed development and nutrients accumulation, we performed a comprehensive global transcriptome analysis using rice tartary buckwheat seeds at different development stages, namely pre-filling stage, filling stage, and mature stage. 24 819 expressed genes, including 108 specifically expressed genes, and 11 676 differentially expressed genes (DEGs) were identified. qRT-PCR analysis was performed on 34 DEGs to validate the transcriptome data, and a good consistence was obtained. Based on their expression patterns, the identified DEGs were classified to eight clusters, and the enriched GO items in each cluster were analyzed. In addition, 633 DEGs related to plant hormones were identified. Furthermore, genes in the biosynthesis pathway of nutrients accumulation were analyzed, including 10, 20, and 23 DEGs corresponding to the biosynthesis of seed storage proteins, flavonoids, and starch, respectively. This is the first transcriptome analysis during seed development of tartary buckwheat. It would provide us a comprehensive understanding of the complex transcriptome dynamics during seed development and gene regulatory mechanism of nutrients accumulation.

## Introduction

Seed is the primary storage organ in plants for storing nutrients such as starch, lipids, and proteins^1^. Therefore, it is of great importance for unraveling the mechanism and regulatory networks during seed development. Seed development in higher plants is a highly complex process. It begins from the double fertilization in ovules, goes through the differentiation and development of various tissues and organs, and finally forms a mature seed^2^.

The processes of seed development and nutrients accumulation are dependent on the expression and regulation of massive expressed genes. Studying the expression patterns of these genes will greatly help us to understand the molecular mechanism of the accumulation of various nutrients during seed development. Transcriptome analyses in seed development of *Arabidopsis* ^2^, rice^1^, wheat^3^, soybean^4^, *Brassica napus* ^5^, and barley^6^ have identified large amounts of related regulatory and functional genes. These genes include transcription factors (TFs)^1,2,4–6^, hormone related genes^1^, genes encoding seed storage proteins (SSPs)^4,5^, genes involved in lipid metabolism^5^, and genes involved in carbohydrate metabolism^5^.

Tartary buckwheat (*Fagopyrum tataricum*) is an annual dicotyledonous crop of *Fagopyrum* Mill. As its origin center, China has the largest planting area and highest yield of tartary buckwheat^7^. Especially in the poor mountain areas of the west of China, tartary buckwheat has gradually become the primary food and economical crop^8^. Tartary buckwheat seed is rich in the primary nutrients, such as starch, fatty acid (linoleic acid), and seed proteins^8–10^. Besides, compared to common buckwheat and cereal crops, tartary buckwheat is richer in the flavonoids (such as rutin and quercetin), dietary fiber, vitamins, chiro-inositol, minerals, and is more balanced in the amino acid composition^11–13^. Therefore, tartary buckwheat is recognized as a particular medicinal and edible plant. Its pharmacological efficiency has been studied massively, including protecting hepatic cells against high glucose-induced oxidative stress and insulin resistance^14^, antioxidant activity^15^, protective effects on high TMAO diet-induced vascular dysfunction and liver injury^16^, inducing G2/M cell cycle arrest and apoptosis^17^.

Massive researches are focused on the contents of various nutrients in tartary buckwheat seeds and their pharmacological efficiency. However, little is known on the transcriptome dynamics and gene regulatory network during seed development of tartary buckwheat. Rice tartary buckwheat is a special type of tartary buckwheat that is with thin shell and easily dehulled. Here we performed a comprehensive global transcriptome analysis using rice tartary buckwheat seeds at different development stages, namely pre-filling stage (PS), filling stage (FS), and mature stage (MS). The expression patterns of genes related to various aspects during seed development were identified. This study would provide a comprehensive understanding of the molecular mechanism of the accumulation of nutrients during seed development at the transcriptional level.

## Results

### Seed development stages and sampling

**Figure 1:**
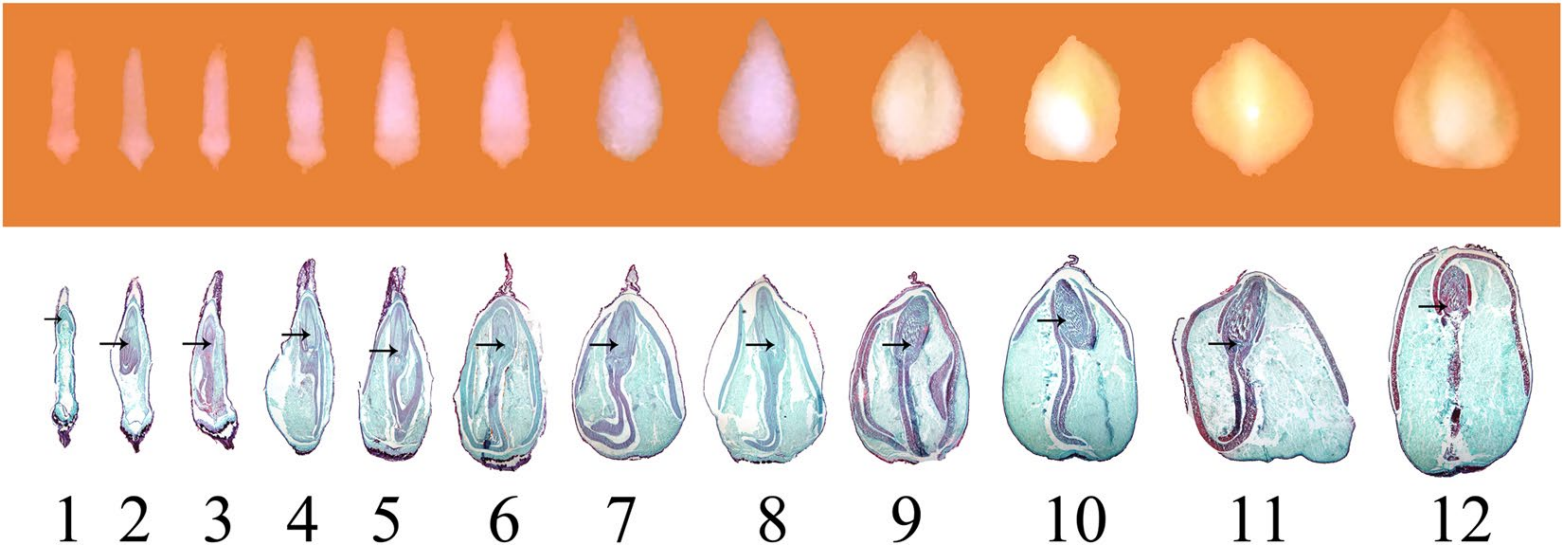
The morphology and their longitudinal sections of tartary buckwheat seeds at different developmental stages. Arrow indicated the embryo in seed.

Tartary buckwheat seeds at 12 developmental stages from their emergence to maturation were selected, and the seed coats were detached. The morphology of the seeds and their longitudinal sections were shown on Fig. 1. At the first three stages, no obvious change was found on their surface, but the embryo gradually developed, from just appearance at the tip of seed (stage 1), to half-length of seed (stage 2), finally growing to full length of seed (stage 3) (embryo was marked by arrow at each stage). Thus, these three stages were named as embryo developing stage or pre-filling stage. From stage 4 to stage 8, seeds plumped up little by little, and the contents of seeds increased as well. Most of the nutrients accumulated at these stages, and they were named as filling stage. From the stage 9 to 12, there seemed no big difference on the longitudinal sections, however, seeds become stronger. At the stage 12, the color of seed coat turned into yellow (figure not shown), which means seeds were maturated completely. The stages from 9 to 12 were named as maturation stage. Next, we chose seeds at these stages for RNA-Seq. Seeds at stage 1 to stage 3 were taken as samples for pre-filling stage (PS). Considering gene expression was usually prior to the nutrients actually accumulation, we just took seeds at stage 4 to stage 6 as samples for filling stage (FS). As seeds have been completely mature and the RNA was degraded at stage 12 (figure not shown), we took seeds at stage 9 to stage 11 as samples for maturation stage (MS). Thus, samples for RNA-Seq were determined.

### High-throughput RNA-Seq and global analysis of gene expression

To have a global view of the seed development at transcriptional level, high-throughput RNA-Seq was performed on RNAs from three seed developmental stages (PS, FS, MS). Three biological replicates at each stage were used for the analysis. Pearson’s rank correlation analysis was performed to evaluate the reproducibility among biological replicates. As shown in Table S1, the correlations between samples among the same biological replicates were good, with a value ranging from 0.983 to 1.000. However, the correlations between samples out of the biological replicates were various, with a value ranging from 0.136 to 0.905. These illustrate that our results were with high reliability and reproducibility of the biological replicates and the sequence method.

After high-throughput RNA-Seq, we obtained 66 107 888 to 81 404 646 raw reads and 62 033 722 to 75 373 688 clean reads for each library. The clean reads were subsequently mapped to the reference genome data of tartary buckwheat. 73.78% to 81.52% clean reads were mapped to the genome and 67.70% to 71.17% clean reads were mapped to the predicted coding sequences of the genome. 24 819 genes were identified in total, with 23 122, 23 067, 23 077, 22 513, 22 550, 22 327, 21 884, 21 772, and 21 692 genes in the libraries of PS1, PS2, PS3, FS1, FS2, FS3, MS1, MS2, and MS3, respectively. Novel transcripts that not included in the reference genes were also identified, ranging from 2 085 to 2 696 for each library (Table 1).

**Table 1:** Summary statistics of RNA-Seq results in seed development of tartary buckwheat.

	**PS1**	**PS2**	**PS3**	**FS1**	**FS2**	**FS3**	**MS1**	**MS2**	**MS3**
Raw reads	72154054	69967156	72154186	71738848	81404646	66107888	72153890	72153878	72155194
Clean reads	66110172	64361106	66046566	66123558	75373688	62033722	65676740	66911478	66514136
Genome map Rate	73.80%	73.78%	74.02%	75.11%	75.18%	76.76%	80.17%	81.52%	81.18%
Gene map Rate	70.98%	71.00%	71.17%	69.65%	68.91%	68.73%	67.70%	69.12%	68.15%
Expressed Gene	23122	23067	23077	22513	22550	22327	21884	21772	21692
Novel Transcripts	2663	2696	2622	2423	2504	2334	2203	2085	2105

**Figure 2:**
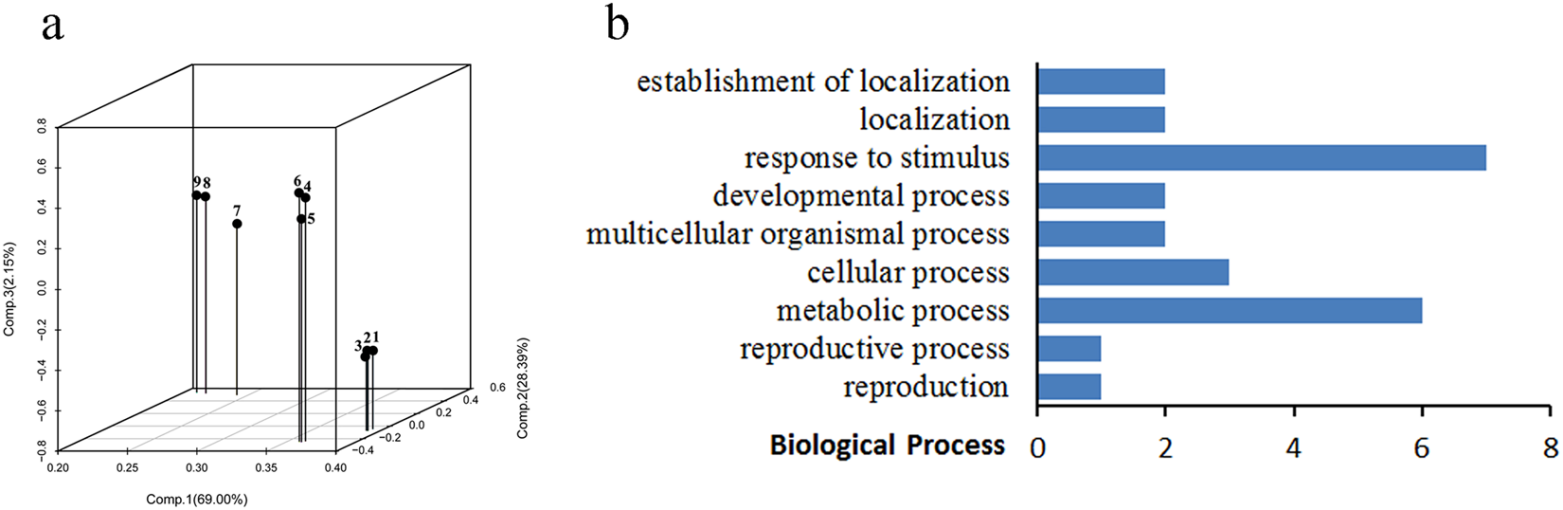
Global analysis of gene expression. (**a**) Principle component analysis (PCA) of all samples during seed development. Samples 1 to 9 represent PS1, PS2, PS3, FS1, FS2, FS3, MS1, MS2, and MS3, respectively. (**b**) Biological process of the stage specifically expressed genes based on GO annotation.

We performed a principle component analysis (PCA) of all samples during seed development. Consistent with their distinct developmental stages, samples from different biological replicates were clustered separately (Fig. 2a). Samples at FS showed closer to PS, rather than MS. This was likely due to only seeds at the early filling stages were selected, and those at later filling stages were ignored. The cluster dendrogram by hclust showed similar results as the PCA analysis (Fig. S1).

In addition, we compared the identified genes at different seed developmental stages. An interesting finding was that 108 genes were specifically expressed at MS (Table S2). Of these, only 26 genes (24%) were successfully annotated to at least one item of GO functions. Based on the sub-categories in biological process, these genes were classified to 9 sub-categories, including response to stimulus (7), metabolic process (6), cellular process (3), multicellular organismal process (2), developmental process (2), localization (2), establishment of localization (2), reproduction (1), and reproductive process (1) (Fig. 2b).

### Differentially expressed genes (DEGs) during seed development

**Figure 3:**
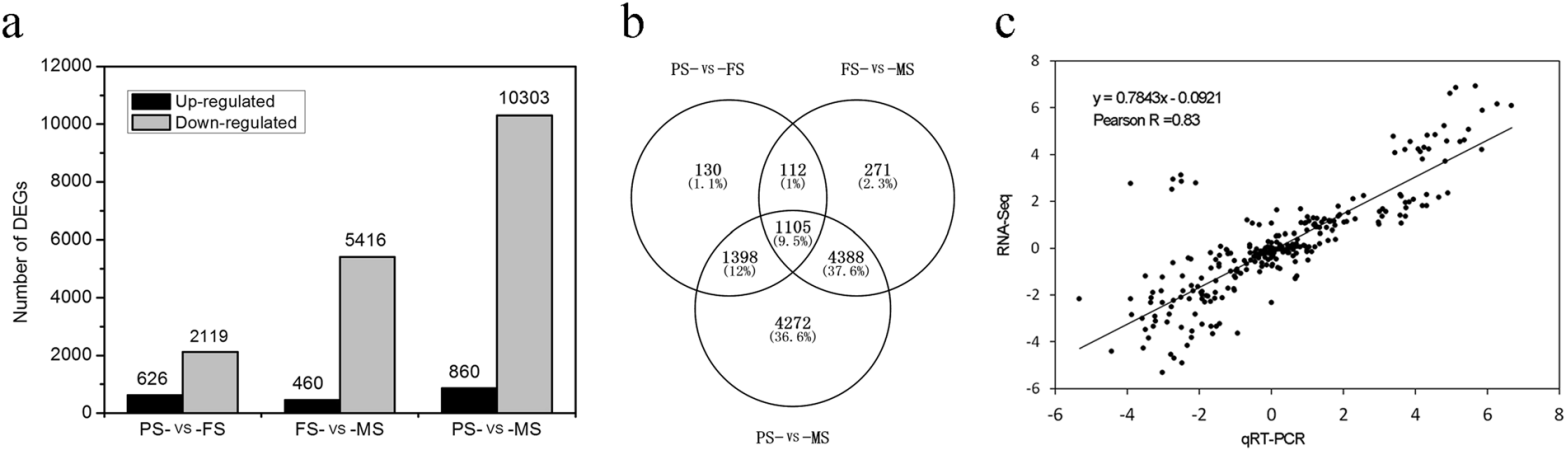
Differentially expressed genes (DEGs) during seed development. (**a**) Statistics of DEGs in PS-vs-FS, FS-vs-MS, and PS-vs-MS, respectively. (**b**) Venn diagram of DEGs in PS-vs-FS, FS-vs-MS, and PS-vs-MS, respectively. (**c**) Validation of the transcriptome data by qRT-PCR.

Using NOISeq, a total of 11 676 DEGs were identified at three stages of seed development (Table S3), among which 2 745, 5 876, and 11 163 DEGs were identified in PS-vs-FS, FS-vs-MS, and PS-vs-MS, respectively (Fig. 3a). Of these DEGs, 1 105 were differentially regulated in all the comparisons of stages with each other, whereas 112, 1 398, and 4 388 were differentially regulated in the comparisons of PS-vs-FS and FS-vs-MS, PS-vs-FS and PS-vs-MS, and FS-vs-MS and PS-vs-MS, respectively. Remaining DEGs were differentially regulated in the comparisons of two stages (Fig. 3b). Besides, qRT-PCR was performed on 34 DEGs that annotated to SSPs biosynthesis (five DEGs), flavonoids biosynthesis (18 DEGs), and hormone biosynthesis and signal pathway (11 DEGs) to validate the transcriptome data. The expression patterns obtained by qRT-PCR were well consistent with those obtained by transcriptome analysis, with a Pearson correlation coefficient of 0.83 (P < 0.0001) (Fig. 3c). In addition, we performed an additional analysis to identify DEGs during seed development of tartary buckwheat, using edgeR. We made a comparison of the results obtained by NOISeq and that obtained by edgeR. We get a good consistency of two methods, with the Pearson correlation coefficient of 0.95, 0.95, and 0.97 in PS-vs-FS, FS-vs-MS, and PS-vs-MS, respectively (Fig. S2).

**Figure 4:**
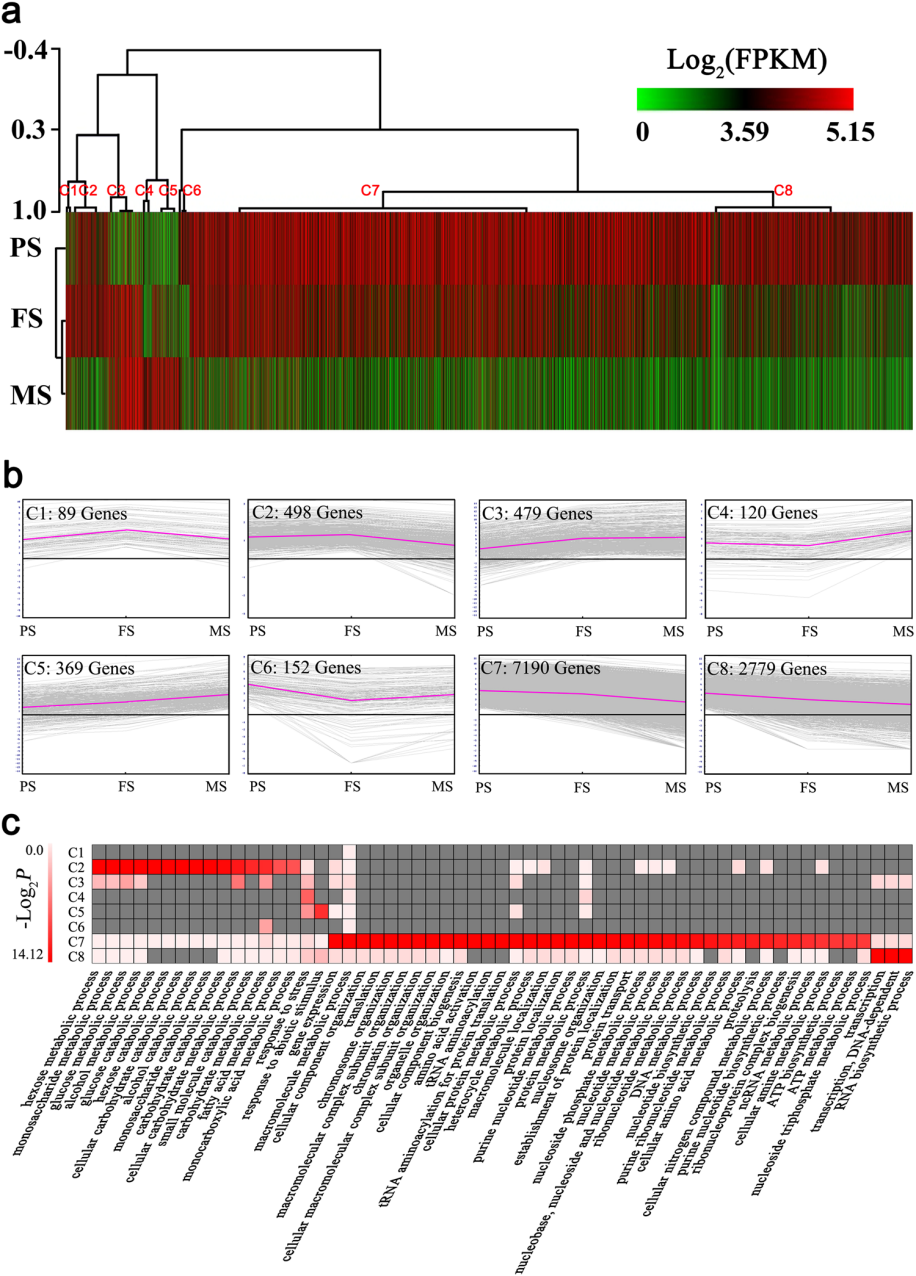
Hierarchical clustering analysis of differentially expressed genes (DEGs) during seed development. (**a**) Hierarchical cluster of the identified DEGs. Eight main clusters were presented as C1 – C8. (**b**) Expression profile of eight clusters correspondance to the Hierarchical cluster result. (**c**) Biological processes of the DEGs significantly enriched in eight clusters, based on GO annotation. The significance was presented as −Log_2_ transformed P-value. Missing GO-slim was represented by grey color.

Hierarchical clustering of all 11 676 DEGs were performed, using the Pearson correlation method associated with average linkage clustering. As a result, eight clusters were identified, namely C1–C8 (Fig. 4a,b). Similarly, they could be divided into two major groups based on the expression patterns. The first group contained C1 to C5, genes in which showed up-regulation patterns. The second group contained C6 to C8, genes in which showed down-regulation patterns. In detail, C1, C2 and C3 were up-regulated at FS, followed by a down-regulation at MS. C4 were up-regulated at MS compared to PS and FS. C5 showed a gradually up-regulation with the seed developmental stage going. C6 were down-regulated at FS and MS compared to PS. C7 and C8 were gradually down-regulated along with seed development. Based on GO annotation, the biological processes at each cluster were analyzed, and 59 significantly enriched biological processes were identified in total, with the FDR ≤ 0.05 (Table S4; Fig. 4c). Among all clusters, C7 (40) and C2 (15) contained most number of enriched biological processes, whereas C1 did not contain any enriched biological process. Generally, numbers of sugar and carbohydrate metabolic and catabolic processes were enriched in C2 and C3, including the metabolic and catabolic processes of hexose, monosaccharide, glucose, and carbohydrate. Fatty acid metabolic process was enriched in C2 as well. Genes in C2 and C3 were higher expressed at FS, suggesting nutrients were massively accumulated at this stage. Genes in C7 and C8 were mostly linked to the various biological processes of biosynthesis and organization, including RNA, ATP, purine nucleotide, and nucleotide biosynthetic processes, ribonucleoprotein complex biogenesis, and cellular component, chromosome, macromolecular complex subunit, cellular macromolecular complex, subunit, organelle, and nucleosome organization. All of these are essential for cell maintenance and growth, but were down regulated during seed development, suggesting maturation was the last step of a seed’s life.

To further understand the metabolic pathways in which the DEGs involved, KEGG pathways were identified (Table S5). The significantly enriched pathways for PS-vs-FS and FS-vs-MS were shown on Table 2. For PS-vs-FS, 1 668 DEGs were successfully annotated to 126 pathways, among which 5 typical nutrients related pathways were significantly enriched (Qvalue < 0.05), including photosynthesis - antenna proteins, photosynthesis, carbon fixation in photosynthetic organisms, cysteine and methionine metabolism, and flavone and flavonol biosynthesis. For FS-vs-MS, 4 013 DEGs were successfully annotated to 133 pathways, among which 10 pathways were significantly enriched (Qvalue < 0.05), including ribosome, proteasome, spliceosome, RNA transport, DNA replication, ribosome biogenesis in eukaryotes, aminoacyl-tRNA biosynthesis, protein processing in endoplasmic reticulum, RNA degradation, and citrate cycle (TCA cycle). Genes in these pathways played important roles on definite processes at stages of seed development.

**Table 2:** Significantly enriched KEGG pathways in seed development of tartary buckwheat (Qvalue ≤ 0.05).

Pathway (PS-VS-FS)	Pathway ID	Gene number	Background number	Pvalue	Qvalue
Photosynthesis - antenna proteins	ko00196	19	22	6.96E-17	8.76E-15
Photosynthesis	ko00195	32	66	9.60E-16	6.05E-14
Carbon fixation in photosynthetic organisms	ko00710	24	109	1.14E-04	4.80E-03
Cysteine and methionine metabolism	ko00270	35	192	2.21E-04	6.98E-03
Flavone and flavonol biosynthesis	ko00944	29	163	1.10E-03	2.76E-02
**Pathway** (**FS-VS-MS**)	**Pathway ID**	**Gene number**	**Background number**	**Pvalue**	**Qvalue**
Ribosome	ko03010	337	487	2.77E-104	3.69E-102
Proteasome	ko03050	50	72	1.84E-16	1.23E-14
Spliceosome	ko03040	121	335	1.17E-07	5.18E-06
RNA transport	ko03013	158	487	3.59E-06	1.19E-04
DNA replication	ko03030	49	115	4.56E-06	1.21E-04
Ribosome biogenesis in eukaryotes	ko03008	81	227	2.27E-05	5.03E-04
Aminoacyl-tRNA biosynthesis	ko00970	46	116	7.73E-05	1.47E-03
Protein processing in endoplasmic reticulum	ko04141	129	431	1.20E-03	1.99E-02
RNA degradation	ko03018	73	227	1.80E-03	2.66E-02
Citrate cycle (TCA cycle)	ko00020	41	117	3.20E-03	4.26E-02

### Phytohormones that regulated during seed development

**Figure 5:**
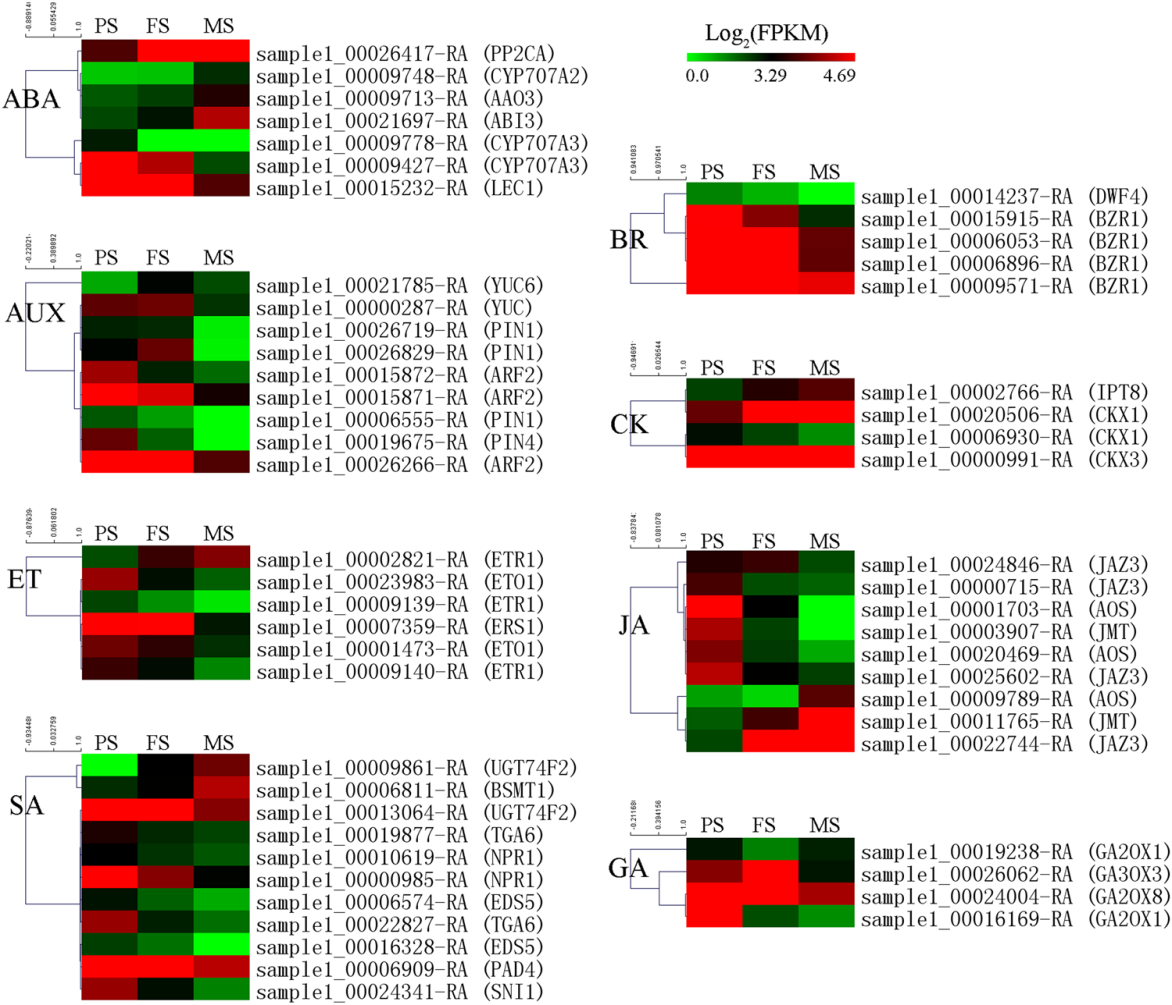
Hierarchical cluster of the hormone related differentially expressed genes (DEGs). ABA, abscisic acid; AUX, auxin; ET, ethylene; SA, salicylic acid; BR, brassinosteroid; CK, cytokinin; JA, jasmonic acid; GA, gibberellin.

**Figure 6:**
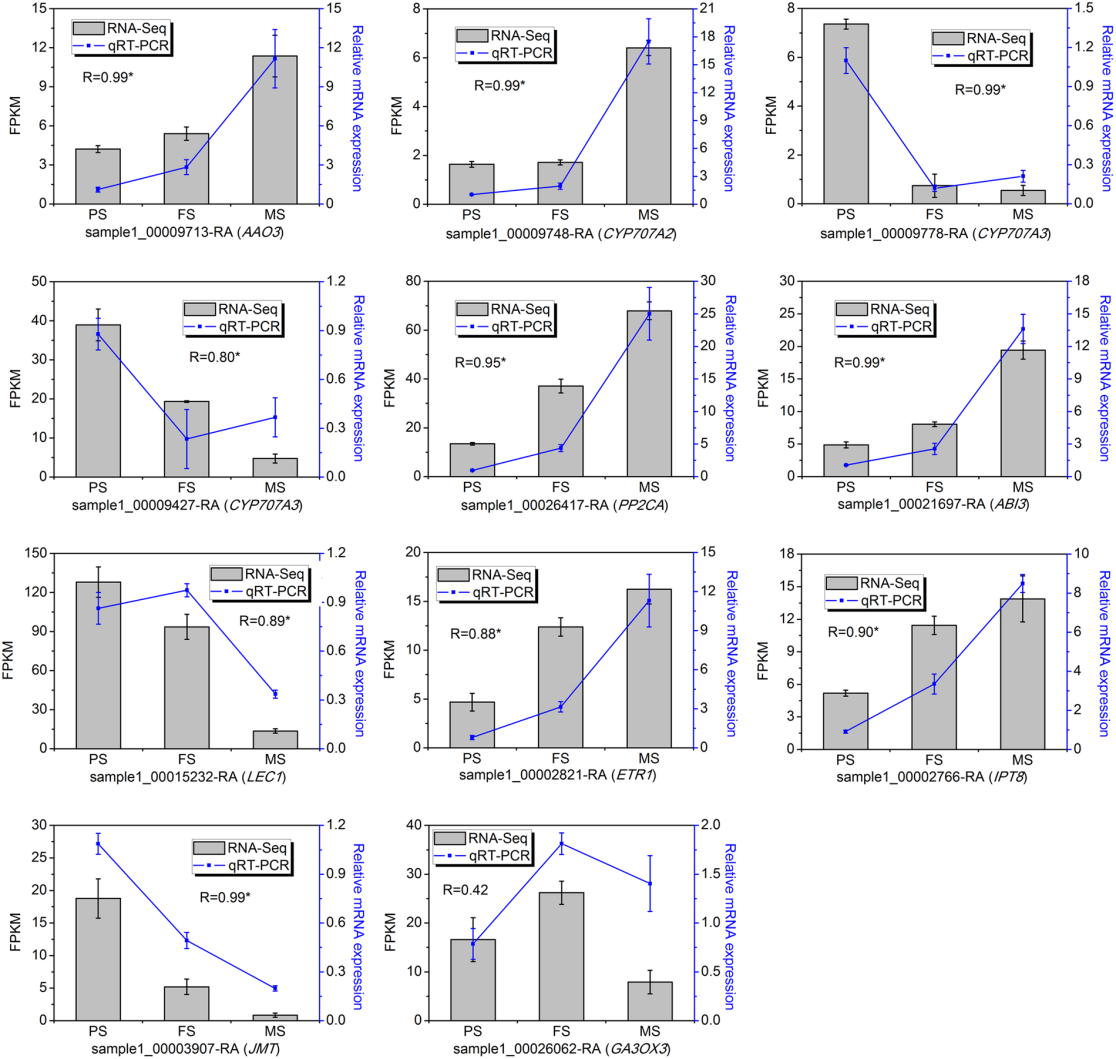
qRT-PCR confirmation of 11 hormone related differentially expressed genes (DEGs).

Phytohormones are reported to be important signals in controlling seed development, maturation, and nutrients accumulation^1^. Therefore, we identified the hormone related DEGs. As a result, 633 DEGs were annotated to genes related to eight major hormones, including abscisic acid (ABA, 149), auxin (AUX, 132), ethylene (ET, 130), salicylic acid (SA, 67), brassinosteroid (BR, 59), cytokinin (CK, 33), jasmonic acid (JA, 33), and gibberellin (GA, 30). These genes were linked to various aspects of plant hormone homeostasis, including biosynthesis (60), metabolism (25), receptor (95), signal transduction (421) and transportion (32) (Table S6, Table 3). Numbers of them that have been found in the complex regulation network of seed development were presented on Fig. 5. Genes involving the pathways of ABA, AUX, ET, SA, BR, CK, JA, and GA could be clustered to two groups dependent on their presented expression patterns. One group was linked to genes that showed up-regulation trends during seed development, whereas the other group was linked to genes that showed down-regulation trends during seed development. In addition, the expression patterns of 11 important hormone related DEGs were verified by qRT-PCR (Fig. 6). These included 7, 1, 1, 1, and 1 DEGs in the biosynthesis and signal pathway of ABA, ET, CK, JA, and GA, respectively. Their expression patterns obtained by qRT-PCR were similar to that obtained by RNA-Seq during seed development, with the exception of sample1_00026062-RA. These differentially expressed hormone related genes suggest that the specific process of seed development of tartary buckwheat might be governed by the regulation of complex phytohormone signal pathways.

**Table 3:** Differentially expressed genes related to eight hormones in seed development of tartary buckwheat.

	Number of DEGs	Biosynthesis	Metabolism	Receptor	Signal transduction	Transportion
ABA	149	7	3	8	131	0
AUX	132	6	4	6	88	28
BR	59	10	2	1	46	0
CK	33	1	3	0	27	2
ET	130	9	7	75	39	0
GA	30	9	0	5	16	0
JA	33	18	0	0	15	0
SA	67	0	6	0	59	2
All	633	60	25	95	421	32

### Expression patterns of the SSP biosynthesis genes during seed development

**Figure 7:**
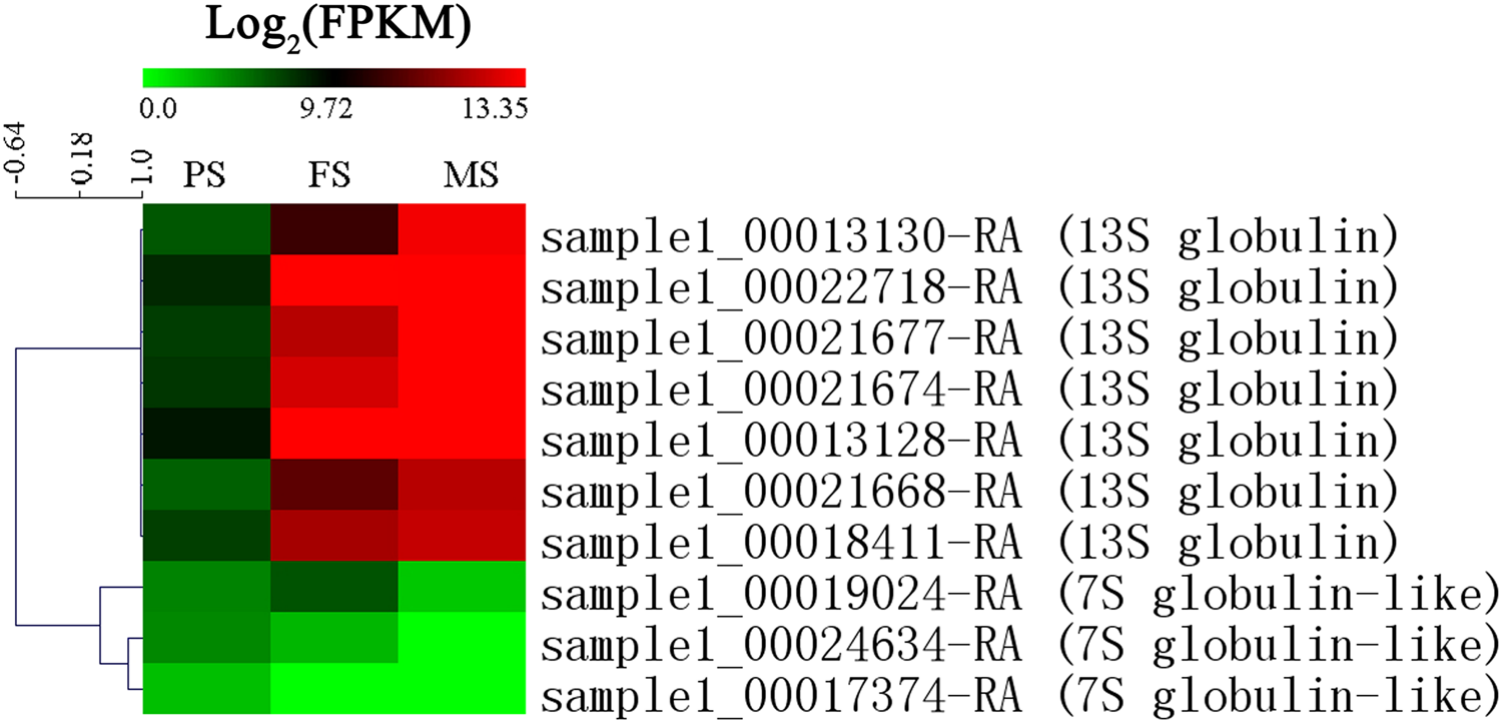
Hierarchical cluster showing the expression patterns of seed storage protein encoding genes that were differentially expressed during seed development.

Tartary buckwheat is famous for its abundant nutrients, including protein, starch, and flavonoids^8,10,18^. Therefore, characterization and analysis of their biosynthesis genes is of great importance. Among the four types of SSPs (globulin, albumin, glutelin, and prolamin), we identified 10 globulin encoding transcripts that were differentially expressed during seed development, including seven 13 S globulin encoding DEGs and three 7 S globulin-like protein encoding DEGs (Fig. 7). Interestingly, all of the seven 13 S globulin encoding DEGs exhibited up-regulation patterns with seed development, whereas three 7 S globulin-like encoding DEGs exhibited down-regulation patterns with seed development. In addition, of the seven 13 S globulin encoding genes, four were annotated to the existing 13 S globulin sequences in common buckwheat (sample1_00022718-RA, sample1_00021677-RA, sample1_00021674-RA, and sample1_00021668-RA), and two were annotated to the existing 13 S globulin sequences in tartary buckwheat (sample1_00013128-RA and sample1_00013130-RA). This indicates that most of the important genes for the accumulation of 13 S globulin during seed development have been found. Except for globulin, genes encoding albumin, glutelin, and prolamin were not significantly changed during seed development in our data.

### DEGs involved in the biosynthesis of flavonoid during seed development

**Figure 8:**
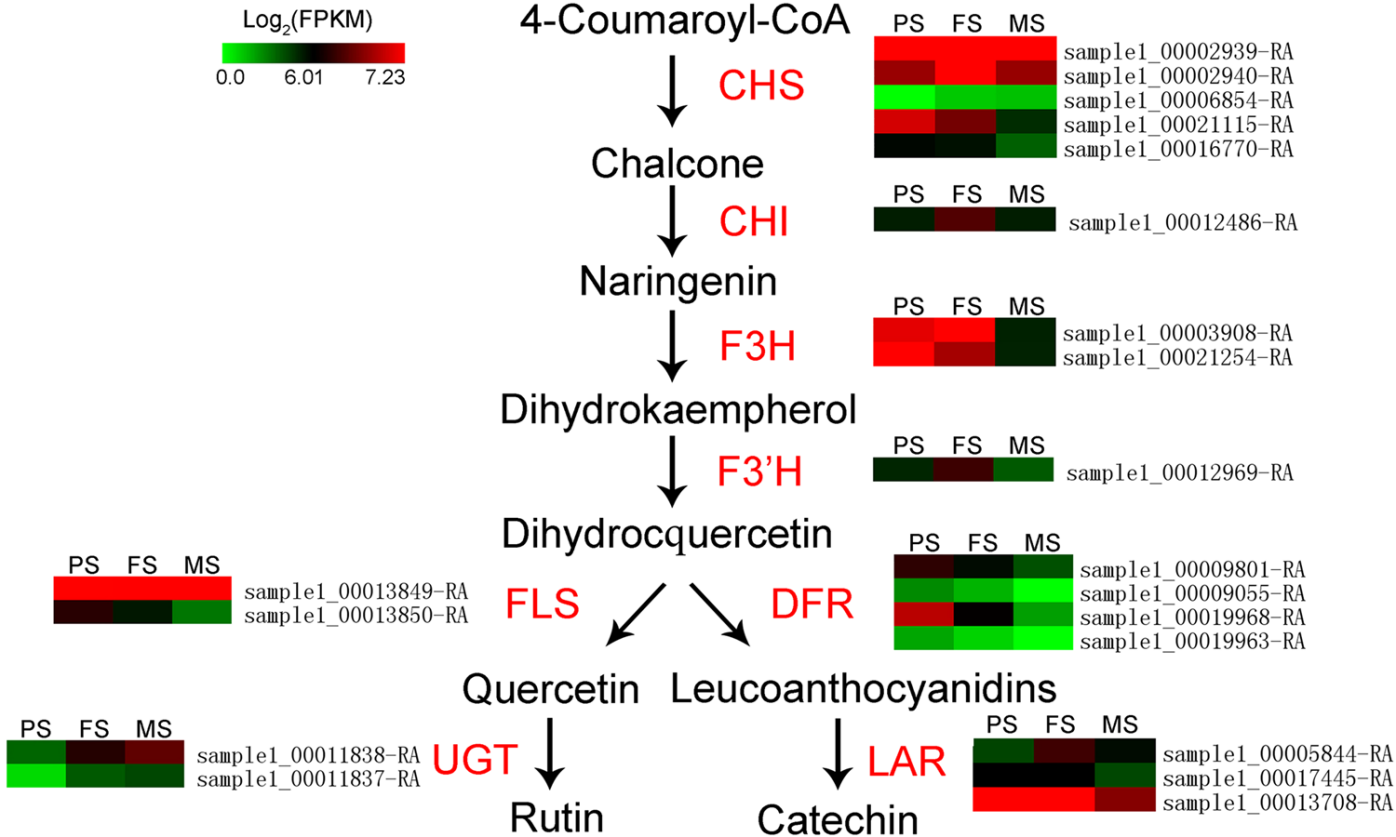
Expression patterns of flavonoid biosynthesis genes that were differentially expressed during seed development.

Tartary buckwheat is rich in flavonoids, including rutin and other flavonoids^12,13,18^. We characterized 20 DEGs that related to flavonoid biosynthesis pathway^19^, including five *chalcone synthase* (*CHS*), one *chalcone isomerase* (*CHI*), two *flavones 3-hydroxylase* (*F3H*), one *flavonoid 3′-hydroxylase* (*F3′H*), two *flavonol synthase* (*FLS*), four *dihydroflavonol-4-reductase* (*DFR*), two *UDP-glycose: glycosyltransferase* (*UGT*), and three *leucoanthocyantin reductase* (*LAR*) (Fig. 8). Five genes encoding *CHS* were differentially expressed during seed development. Among them, the expression of sample1_00002939-RA and sample1_00002940-RA were increased at FS, followed by a decrease at MS; sample1_00006854-RA was up-regulated along with seed development; whereas sample1_00021115-RA and sample1_00016770-RA were gradually down-regulated along with seed development. Sample1_00012486-RA, orthologous to *CHI*, was up-regulated at FS and followed by a down-regulation at MS. Two genes encoding F3H exhibited two different expression patterns: sample1_00003908-RA was up-regulated at FS but later down-regulated at MS, whereas sample1_00021254-RA was down-regulated with seed development. One *F3′H*, sample1_00012969-RA, whose expression level was firstly increased at FS but later decreased at MS, was also identified. Two genes encoding FLS showed two different expression patterns: sample1_00013849-RA was up-regulated at FS but later down-regulated at MS, whereas sample1_00013850-RA was down-regulated with seed development. Four genes encoding DFR were identified (sample1_00019968-RA, sample1_00009801-RA, sample1_00009055-RA, and sample1_00019963-RA), with similar expression trend that down-regulated with seed development. Two UGT encoding genes were also identified (sample1_00011838-RA and sample1_00011837-RA), with up-regulation pattern during seed development. In addition, we identified three *LAR* genes and they showed two expression patterns: sample1_00005844-RA was up-regulated at FS but down-regulated at MS; sample1_00017445-RA and sample1_00013708-RA had higher expression level at PS and FS, but were down-regulated at MS. Overall, most of DEGs in flavonoid biosynthesis pathway showed a higher expression at PS and MS, suggesting the biosynthesis of tartary buckwheat flavonoids occurred before seed maturation.

### Identification of starch biosynthesis genes differentially regulated during seed development

**Figure 9:**
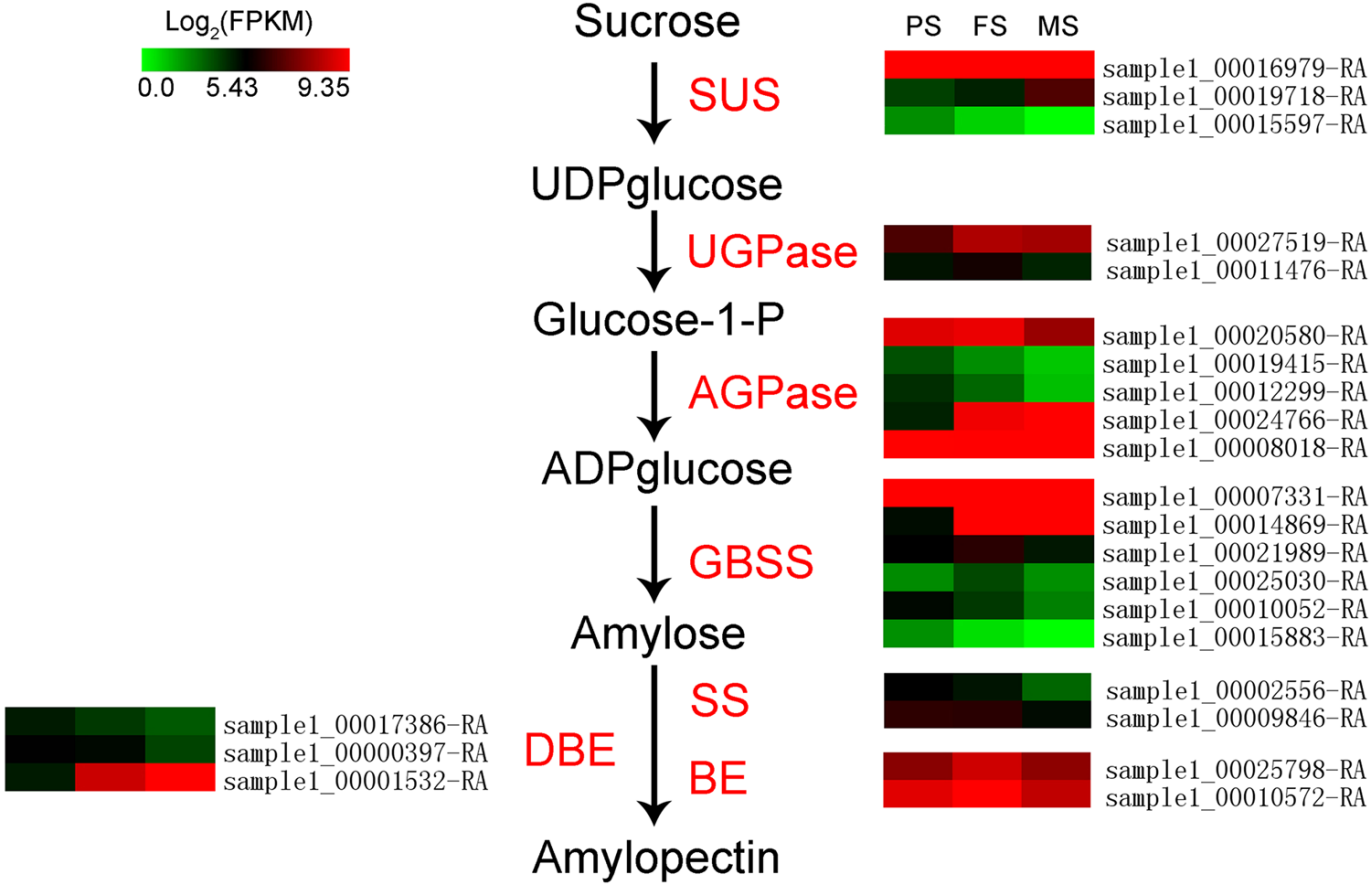
Expression patterns of starch biosynthesis genes that were differentially expressed during seed development.

Starch accumulation is an essential process for seed development. We identified 23 DEGs involved in the starch biosynthesis pathway, including *sucrose synthase* (*SUS*), *UDP glucose pyrophosphorylase* (*UGPase*), *ADP glucose pyrophosphorylase* (*AGPase*), *granule bound starch synthase* (*GBSS*), *starch synthase* (*SS*), *starch-branching enzyme* (*BE*), and *debranching enzyme* (*DBE*) (Fig. 9). Three *SUS* genes were differentially expressed, of which, sample1_00016979-RA was up-regulated at FS comparing to PS, and followed by a down-regulation at MS; sample1_00019718-RA was up-regulated with seed development stages; sample1_00015597-RA was down-regulated with seed development stages. Two *UGPase* genes, sample1_00027519-RA and sample1_00011476-RA, were up-regulated at FS but followed by a down-regulation at MS. Five AGPase encoding genes showed three different expression patterns: sample1_00019415-RA and sample1_00012299-RA were down-regulated with seed development stage going; sample1_00024766-RA was up-regulated with seed development stage going; whereas sample1_00020580-RA and sample1_00008018-RA were firstly up-regulated at FS but down-regulated at MS. Six *GBSS* genes also showed three different expression patterns: sample1_00010052-RA and sample1_00015883-RA were down-regulated with seed development stage going; sample1_00007331-RA and sample1_00014869-RA were up-regulated with seed development stage going; whereas sample1_00021989-RA and sample1_00025030-RA were firstly up-regulated at FS but down-regulated at MS. Two *SS* genes, sample1_00002556-RA and sample1_00009846-RA, were down-regulated with seed development. Two *BE* genes, sample1_00025798-RA and sample1_00010572-RA were up-regulated at FS but down-regulated at MS. At last, three *DBE*s showed definitely opposite expression patterns: sample1_00017386-RA and sample1_00000397-RA were down-regulated with seed development whereas sample1_00001532-RA was up-regulated with seed development.

## Discussion

### Identification of stage specifically expressed genes

Based on our analysis, 108 specifically expressed genes involved in 9 sub-categories of GO biological process were identified at MS (Table S2; Fig. 2b). Some of them were annotated to the well-defined genes whose functions have been characterized. For example, fagopyritols are accumulated in the embryos of mature seeds in *F*. *esculentum* ^20^, and three genes for their biosynthesis have been identified, namely *fagopyritol synthase* 1 *(FeGolS-1*), *FeGolS-2*, and *FeGolS-3* ^21^. In our result, sample1_00011242-RA, annotated to *FeGolS-2* of *F*. *esculentum*, was specifically expressed at MS. This suggests fagopyritols in tartary buckwheat are also accumulated in the embryos of mature seeds, similar to that in common buckwheat. One SSP encoding gene, sample1_00013130-RA, was specifically expressed at MS. It encodes a 13 S globulin in tartary buckwheat, suggesting an accumulation of 13 S globulin at MS of seeds. Function of MADS-box TFs in seed development have been found in several species, including *Arabidopsis* ^22^, rice^23^, and tomato^24^. We identified a MADS-box gene (sample1_00005419-RA) that specifically expressed at MS, suggesting it has also a role in seed maturation of tartary buckwheat. In addition, two specifically expressed genes were related to plant hormone biosynthesis (sample1_00006058-RA and sample1_00009789-RA). Sample1_00006058-RA was homologous to *gibberellin 20-oxidase*. Gibberellin 20-oxidase catalyze C20-GA substrates to C19-GA products, and is necessary for the formation of bioactive GA^25^. Allene oxide synthase (AOS) is important in the JA biosynthesis pathway^26^. One homolog of *AOS*, sample1_00009789-RA, was also specifically expressed at MS. Furthermore, some specifically expressed genes were related to plant defense responses, including three genes encoding cytochrome P450^27^ (sample1_00019807-RA, sample1_00017522-RA, and sample1_00026585-RA), three genes encoding heat shock protein^28^ (sample1_00015507-RA, sample1_00019961-RA, and sample1_00021423-RA), and one gene belonging to HSF TF family^29^ (sample1_00018030-RA).

### Cluster of DEGs during seed development

Base on the expression patterns of DEGs, we clustered them to eight clusters, namely C1 – C8 (Fig. 4a,b). They could be roughly divided to two groups: the up-regulation group (C1 to C5) and the down-regulation group (C6 to C8). The number of DEGs in the down-regulation group was several times more than that in the up-regulation group, suggesting more inactive events are occurred in the progress of seed development than the active events. This is further elucidated by the enriched biological processes based on GO annotation (Table S4; Fig. 4c). Massive genes linked to cell maintenance and organization were included in the down-regulation group. All of these were essential for cell stabilization and growth, but were down regulated during seed development, suggesting although the last step morphologically of a seed’s life is maturation and desiccation, their corresponding molecular events occur far earlier, which begin at the completion of embryo development (PS). The decrease of genes related to cell maintenance and energy as the cotyledons approached the mature was also found in soybean^4^. The active events occurs during seed development are more biologically particular and meaningful for seeds. This is demonstrated by the biological processes involving genes in the up-regulation group, including numbers of carbohydrate and fatty acid metabolic processes which are correlated to the nutrients accumulation and storage in seeds, leading seeds to maturation^30^.

### Hormone signals involved in seed development

We identified 633 DEGs that in the hormone biosynthesis and signal pathway, including 149 ABA, 132 AUX, 130 ET, 67 SA, 59 BR, 33 CK, 33 JA, and 30 GA related genes during tartary buckwheat seed development (Table 3; Figs 5 and 6). Most hormone related DEGs identified were linked to ABA. ABA is a key hormone required for seed development^31–33^. Previous study has revealed that *CYP707A* in ABA catabolism has high expression level throughout seed development in bean^31^. Three homologs of the *CYP707A* were differentially regulated in our data (sample1_00009748-RA, sample1_00009778-RA, sample1_00009427-RA), suggesting that ABA 8’-hydroxylases during ABA catabolism may also occur in seed development of tartary buckwheat. *PP2C* has a major role in the ABA response in seeds, and its expression is increased during late seed maturation^32^. Its homolog in tartary buckwheat was also up-regulated as seed development (sample1_00026417-RA). *LEC1* and *ABI3* are two other genes that connect to seed maturation in ABA signaling^33^, and their homologs were differentially expressed during seed development in tartary buckwheat (sample1_00021697-RA and sample1_00015232-RA). In addition, a homolog of the essential gene in ABA biosynthesis, *AAO3*, was identified and exhibited an up-regulation during tartary buckwheat seed development (sample1_00009713-RA).

AUX is also necessary for seed development. Most of the studies focus on its role in determining seed size, with the influence of the expression of gene in AUX biosynthesis (*ZmTar3*, *ZmTar1*, and *ZmYuc1*) and signaling (auxin efflux carriers, *PIN*; *ARF2*)^34,35^. Homologs of *ZmYuc1* (sample1_00021785-RA and sample1_00000287-RA*)*, *PIN* (sample1_00006555-RA, sample1_00026719-RA, sample1_00026829-RA, sample1_00019675-RA), *ARF2* (sample1_00015872-RA, sample1_00015871-RA, and sample1_00026266-RA) were significantly differentially expressed during tartary buckwheat seed development. Most of these DEGs had higher expression level at PS and FS, this is correspondent to that cell division and expansion is faster to form larger size seeds at these stages.

The molecular evidence illustrate ethylene is in the complex regulation of seed size and seed shape, in which genes in ethylene biosynthesis (*EIN2*, *ERS1*, *ETR1*), signaling (*CTR1*, *ETO1*, *ETR1*, *EIN2*), and catabolism (*ACC deaminase*) are involved^36,37^. Among the 130 ET related DEGs we identified, six were homologous to the characterized genes regulating seed development. Two homologs of *ETO1* (sample1_00001473-RA and sample1_00023983-RA) were down-regulated as seed development. One homologs of *ERS1* (sample1_00007359-RA) was down-regulated as seed development as well. Three homologs of *ETR1* (sample1_00002821-RA, sample1_00009139-RA and sample1_00009140-RA) exhibited two opposite expression patterns: sample1_00002821-RA was up-regulated with seed development; whereas sample1_00009139-RA and sample1_00009140-RA were down-regulated with seed development.

The effects of SA on seed development exhibit two distinct opinions. On one hand, exogenous application of SA would increase seed’s dry mass, and pod and seed number^38^. On the other hand, however, SA delays fruit ripening, and SA deficiency mutants increase seed yield and antioxidant vitamin concentration^39^. Although no SA biosynthesis DEGs was identified in our data, three homologs in SA catabolism, and 8 genes in SA signal were identified. These included two DEGs homologous to *UGT74F2* (sample1_00013064-RA and sample1_00009861-RA), one DEGs homologous to *BSMT1* (sample1_00006811-RA), one DEGs homologous to *PAD4* (sample1_00006909-RA), two DEGs homologous to *EDS5* (sample1_00016328-RA and sample1_00006574-RA), two DEGs homologous to *NPR1* (sample1_00000985-RA and sample1_00010619-RA), two DEGs homologous to *TGA6* (sample1_00019877-RA and sample1_00022827-RA), and one DEGs homologous to *SNI1* (sample1_00024341-RA). They showed two opposite expression patterns: parts of them were up expressed during seed development, whereas the rest were down expressed during seed development.

BR regulates embryo development, seed size, seed shape, seed length, and seed yield^40^. The characterized genes include *BZR1*, *BES1*, *DWF4*, and *DWAFR11*. Five of their homologs were identified in our data, including one in BR biosynthesis(sample1_00014237-RA, homolog of *DWF4*) and four in BR signal pathway (sample1_00015915-RA, sample1_00006053-RA, sample1_00006896-RA, and sample1_00009571-RA, homologs of *BZR1*). All of their expression levels were gradually falling down with seed development, suggesting BR might regulate seed development at the early stage.

CK is also reported to function on seed development, such as seed size, seed yield, embryonic growth, with the involvement of genes encoding isopentenyl transferase (*IPT*), cytokinin oxidase/dehydrogenase (*CKX*), and histidine kinase (*AHK*)^41^. Four DEGs of the homologous genes were identified, including one homolog of *IPT8* (sample1_00002766-RA), two homologs of *CKX1* (sample1_00006930-RA and sample1_00020506-RA), one homolog of *CKX3* (sample1_00000991-RA). Sample1_00002766-RA and sample1_00020506-RA were up-regulated during seed development, whereas sample1_00006930-RA and sample1_00000991-RA were down-regulated during seed development. This suggests they may have different functions in regulating seed development of tartary buckwheat.

JA related processes in seed development contain embryo development, fruit ripeness, nutrients compositions (proximates, amino acids, fatty acids, isoflavones, and antinutrients), and seed production. These are accompanied with the expression of numbers of JA-inducible genes, including *OPDA*, *AOS1* and *JAZs*, *JMT* ^42^. Nine homologs of them were identified in our data, of which three DEGs were homologous to *AOS* (sample1_00009789-RA, sample1_00020469-RA, sample1_00001703-RA), two were homologous to JMT (sample1_00003907-RA, sample1_00011765-RA), four were homologous to *JAZ3* (sample1_00024846-RA, sample1_00000715-RA, sample1_00025602-RA, sample1_00022744-RA). All of them but one showed down-regulation with seed development, suggesting that JA might also be at the early regulatory network of seed development stages in tartary buckwheat.

Previous studies have revealed that genes encoding GA 2-oxidase and GA 3-oxidase in GA biosynthesis pathway can affect seed development, starch biosynthesis, embryo and seed coat development^43,44^. Four homologs of these genes, sample1_00019238-RA, sample1_00026062-RA, sample1_00024004-RA, and sample1_00016169-RA, were identified, all of which showed down-regulation expression as seed development.

### Accumulation of the nutrients related genes during seed development of tartary buckwheat

Tartary buckwheat is famous for its abundant nutrients, including proteins, starch, and flavonoids^8,10,18^. Therefore, characterization and analysis of their biosynthesis genes is of great importance. SSPs are the main buckwheat allergen, and tens of their encoding sequences have been reported, mostly in common buckwheat^45,46^. In tartary buckwheat, three SSPs have been identified, including a 16-kDa major allergen belonged to 2S albumin^47^, a 24 kDa allergenic protein homology with a legumin-like protein^48^, and an allergen with high similarity to legume-like 13S globulin storage protein^49^. In our transcriptome, we identified 10 DEGs encoding 13S globulins (7) and 7S globulin-like proteins (3) (Fig. 7). All of the seven 13S globulin encoding DEGs exhibited up-regulation patterns with seed development. And six of them were matched to the existing buckwheat 13S globulin sequences. Sample1_00021677-RA, sample1_00021674-RA and sample1_00021668-RA were matched to a 13S globulin of common buckwheat (GenBank: BAO50862.1). Sample1_00022718-RA was also matched to a 13S globulin of common buckwheat (GenBank: BAO50870.1). Sample1_00013128-RA and sample1_00013130-RA were matched to a 13S globulin of tartary buckwheat (GenBank: ABI32184.1). Our transcriptome data is in accordance with previous reports that 13S globulin is one of the most dominant allergens, and accounts for 43% of total seed proteins in buckwheat seeds^46^.

Flavonoids, especially rutin, are the most widely studied nutrients in buckwheat^13,50^. Comparing with common buckwheat, the content of rutin in tartary buckwheat is higher^11^. At the transcriptome level, genes in the flavonoid biosynthesis have been thoroughly characterized and analyzed in buckwheat^19,50–52^. As shown on Fig. 8, 20 DEGs involved in flavonoid biosynthesis were identified. Most of them in the upstream of flavonoid biosynthesis pathway exhibited higher expression at PS and MS. However, two UGT encoding genes that catalase quercetin substrate to rutin, sample1_00011838-RA and sample1_00011837-RA, were up-regulated during seed development, consistence with rutin is the main accumulated flavonoid at matured buckwheat seeds^50^. In addition, the flavonoid biosynthesis genes were mostly reported in flower^19,51,52^. This is largely due to the inflorescence stage has the highest rutin content in all of the buckwheat species^50^. In tartary buckwheat and rice tartary buckwheat, matured seeds have the second highest rutin content^50^. Our results will provide molecular evidence for previous study, as well as candidate genes that primarily participate in the seed flavonoid biosynthesis of tartary buckwheat for further research.

Starch is the major form of carbohydrates accumulated at mature seeds and the main nutrient that make the seeds and other storage organs expand and enlarge. Genes in the starch biosynthesis pathway have been identified entirely, involving *SUS*, *UGPase*, *AGPase*, *GBSS*, *SS*, *BE*, and *DBE* ^53^. In buckwheat, starch account for over 70% of the seeds’ dry weight, thus lead its quality to be the main determinant of the quality of buckwheat seeds production^8^. However, the buckwheat starch biosynthesis genes remain largely unknown, except for two *GBSS* genes^54,55^. In our data, we identified 23 genes in starch biosynthesis which were differentially regulated during seed development of tartary buckwheat, including 3, 2, 5, 6, 2, 2, and 3 corresponding to the genes encoding SUS, UGPase, AGPase, GBSS, SS, BE, and DBE, respectively (Fig. 9). Of these, sample1_00010052-RA is matched to the *GBSSI* gene in tartary buckwheat (AHA36967.1). GBSS, also known as waxy protein, is the most direct enzyme that influences amylose content and starch quality^53^. The identified starch biosynthesis DEGs provide expression patterns for starch biosynthesis genes in seed development of tartary buckwheat, and would accelerate function analysis of them as well.

## Conclusions

We performed a comprehensive global transcriptome analysis at seed developmental stages (PS, FS, and MS) of rice tartary buckwheat. Totally, 24 819 expressed genes were identified, including 108 genes that were specifically expressed at MS; and 11 676 DEGs were identified. The transcriptome data was confirmed by a qRT-PCR analysis on 34 DEGs, and a good consistence was obtained. All DEGs were classified into eight clusters based on their expression patterns. The GO annotation and KEGG pathways were also enriched for these DEGs. In addition, 633 hormone-related DEGs were also identified, which would be the key regulators at different stages of seed development of tartary buckwheat. Furthermore, 10 DEGs encoding SSPs, 20 DEGs involving in flavonoid biosynthesis, and 23 DEGs in starch biosynthesis were identified, suggesting the expression patterns of the nutrients accumulation related genes during seed development of tartary buckwheat. To our knowledge, this is the first study that revealed a global transcriptomic dynamics during seed development in tartary buckwheat. It would help us to understand the complex transcriptome dynamics and gene regulatory mechanism during the process of seed development and nutrients accumulation.

## Materials and Methods

### Plant materials and paraffin-embedded sections

A thin seed shell, high yield new line, Gui Miku 1503–11 (simply Mi 11) was used as our material, which is derived from the cross progenies of tartary buckwheat (thick shell) Jinqiaomai 2 native to Shanxi and local rice tartary buckwheat (thin shell) Xiaomiqiao native to Yunnan. It was planted in our testing field in spring, and with normal field management during the growth periods. Seeds were taken at 12 developmental stages from their emergence to maturation after pollination. Then seed coats were stripped and the remaining seeds were fixed in formalin-aceto-alcohol solution immediately for paraffin-embedded sections. Making and observation of the paraffin-embedded sections was performed as previous described procedure^56^. Based on the morphology of the seeds and their longitudinal sections, samples for RNA-Seq were collected at three stages, namely PS (corresponding to stage 1 to stage 3), FS (corresponding to stage 4 to stage 6), and MS (corresponding to stage 9 to stage 12) (see Results for details). For RNA extraction, the materials were frozen in liquid nitrogen immediately. Seed coats were stripped on a mixture of ice and liquid nitrogen, and the remaining seeds were stored at −80 °C.

### RNA extraction, librayry construction, and sequencing

RNA was extracted using plant RNA purification kit that containing Dnase I (TianGen, Beijing, China), according to the manufacturer’s instruction. RNA samples with A260/280 ≥ 1.8, A260/230 ≥ 1.8, RIN ≥ 6.5, and 28 S/18 S ≥ 1.0 in quality, and concentration ≥20 ng/μL and total RNA amount ≥2 μg in quantity was acceptable for next step experiment, including library construction and qRT-PCR analysis. Samples from each stage consisted of three biological replicates, thus coming to nine RNA samples. All of the RNA samples were used to construct cDNA libraries for transcriptome sequencing.

For library construction, 2 μg RNA was used for each sample. mRNA was purified using Dynabeads mRNA purification kit in accordance to the manufacturer’s instruction(Invitrogen). Then the mRNA was cleaved to 200–250 bp small fragments in Fragment buffer. First strand cDNA was then generated using these small fragments by N6 primers, and second strand cDNA was generated by Second Strand Master Mix. Then cDNA was purified using QIAquick PCR Purification Kit (QIAGEN), combined with End Repair Mix, and add with A-tailing using A-Tailing Mix. To select cDNA with 300–350bp size, whole cDNA was used to perform a 2% agarose gel electrophoresis, and then purified the right size cDNA using QIAquick Gel Extraction kit (QIAGEN). PCR amplification was subsequently carried out using PCR Primer Cocktail to enrich the cDNA fragments, and the PCR products were purified using Ampure XP Beads (AGENCOURT). The quality of the libraries were determined by Agilent 2100 bioanalyzer instrument (Agilent DNA 1000 Reagents) and qRT-PCR (TaqMan Probe).

At last, the qualified libraries were amplified to generate cluster using TruSeq PE Cluster Kit V3-cBot-Hs (Illumina). Then they were sequenced on the Illumina HiSeq. 4000 System with read length of 101 bp and paired-end method. All of the raw data generated by sequencing have been deposited in NCBI SRA under Accession: PRJNA352888.

### Mapping and analysis of DEGs

Trimmomatic was used to obtain clean reads. Raw data were filtered by removing adapter containing reads, reads with N (unknown base) percentage more than 10%, and low quality reads. Then clean reads were mapped to the reference genome data of tartary buckwheat (SRA accession number: PRJNA395279), using TopHat2. All of the gene sequences analyzed in this manuscript were presented in Table S7. These include 108 stage specifically expressed genes, 633 hormone related genes, 10 SSP encoding genes, 20 flavonoid biosynthesis involving genes, and 23 starch biosynthesis involving genes. The expressed genes were normalized and quantified as FPKM value, using RSEM^57^. The cluster of samples was analyzed using Euclidean distance by ward method in R language. All of the detected genes were annotated based on NR database, GO database, and KEGG database, with the e value of 1e-5.

DEGs were identified using two packages: 1) NOISeq package, with the threshold of absolute value of log_2_Ratio ≥ 1 and Probability ≥ 0.8^58^; 2) edgeR package, for an additional analysis to confirm the DEGs obtained by NOISeq. Hierarchical clustering of DEGs was performed using the Pearson correlation method associated with average linkage clustering by MeV. GO enrichment was performed using agriGO. KOBAS 2.0 was then used to identify the significantly enriched metabolic pathways. And a local blastp was performed with the AHD2.0 to identify plant hormone related genes. The threshold for blast was set as 1e-5.

### qRT-PCR analysis

qRT-PCR analysis was performed to verify transcriptome results. RNA samples used for qRT-PCR were identical to those used for the RNA-Seq. 34 DEGs were selected, and gene-specific primers were designed using Primer Premier 6.0 (Table S8). *Actin* was used as the inner reference gene. qRT-PCR was carried out using SYBR^®^ Premix Ex Taq^™^ II (Tli RNaseH Plus) (RR820b, TAKARA, DaLian, China) on an ABI ViiA^™^ 7 real-time PCR system (Applied Biosystems, USA) with three technical replicates. Amplification reactions were initiated with a denaturing step (95 °C for 10 min), followed by 40 cycles of denaturing (95 °C for 10 s), annealing (60 °C for 30 s) and extension (72 °C for 40 s). Data were analyzed by 2^−(∆∆Ct)^ method to obtain relative mRNA expression data.

## Electronic supplementary material

Supplementary Information

Table S3

Table S5

Table S6

Table S7
