# Supplementary material for: Global transcriptome analysis and identification of genes involved in nutrients accumulation during seed development of rice tartary buckwheat (Fagopyrum Tararicum)
Source: Sci Rep. 2017 Sep 18;7:11792. doi: 10.1038/s41598-017-11929-z (PMC5603606; doi:10.1038/s41598-017-11929-z)
Supplement: Supplementary file 1 — Supplementary Information [file 41598_2017_11929_MOESM1_ESM.pdf]

Supplementary Information

Global transcriptome analysis and identification of genes involved in nutrients accumulation during seed development of rice tartary buckwheat (*Fagopyrum Tararicum*)

Juan Huang[1], Jiao Deng[1], Taoxiong Shi[1], Qijiao Chen[1], Chenggang Liang[1], Ziye Meng[1], Liwei Zhu[1], Yan Wang[1], Fengli Zhao[2], Shizhou Yu[3] & Qingfu Chen[1]*

1 Research Center of Guizhou Buckwheat Engineering and Technology, Research Center of Buckwheat Industry Technology, Guizhou Normal University, Baoshan Beilu 116, Guiyang 550001, Guizhou, P.R. China.
2 Agricultural Genomics Institute, Chinese Academy of Agricultural Sciences, Pengfei Road No. 7, Dapeng New District, Shenzhen 518120, Guangdong, P.R. China
3 Guizhou Academy of Tobacco Science, Guiyang 550081, Guizhou, P.R. China.

***Corresponding author:**
Qinfu Chen
Email: cqf1966@163.com

List of Supplementary Materials

Fig. S1. Cluster dendrogram of nine libraries by hclust method.
Fig. S2. Comparison of the DEGs obtained by NOISeq and the DEGs obtained by edgeR.

Table S1. Pearson's rank correlation of nine libraries.
Table S2. List of the stage specifically expressed genes.
Table S3. List of the differentially expressed genes during seed development of tartary buckwheat.
Table S4. Biological processes of the DEGs significantly enriched based on GO annotation (FDR ≤0.05).
Tasble S5. List of the KEGG pathways in PS-vs-FS (a) and FS-vs-MS (b).
Table S6. List of hormone related genes differentially expressed during seed development.
Table S7. All of the gene sequences analyzed in this study.
Table S8. List of primers for qRT-PCR analysis.

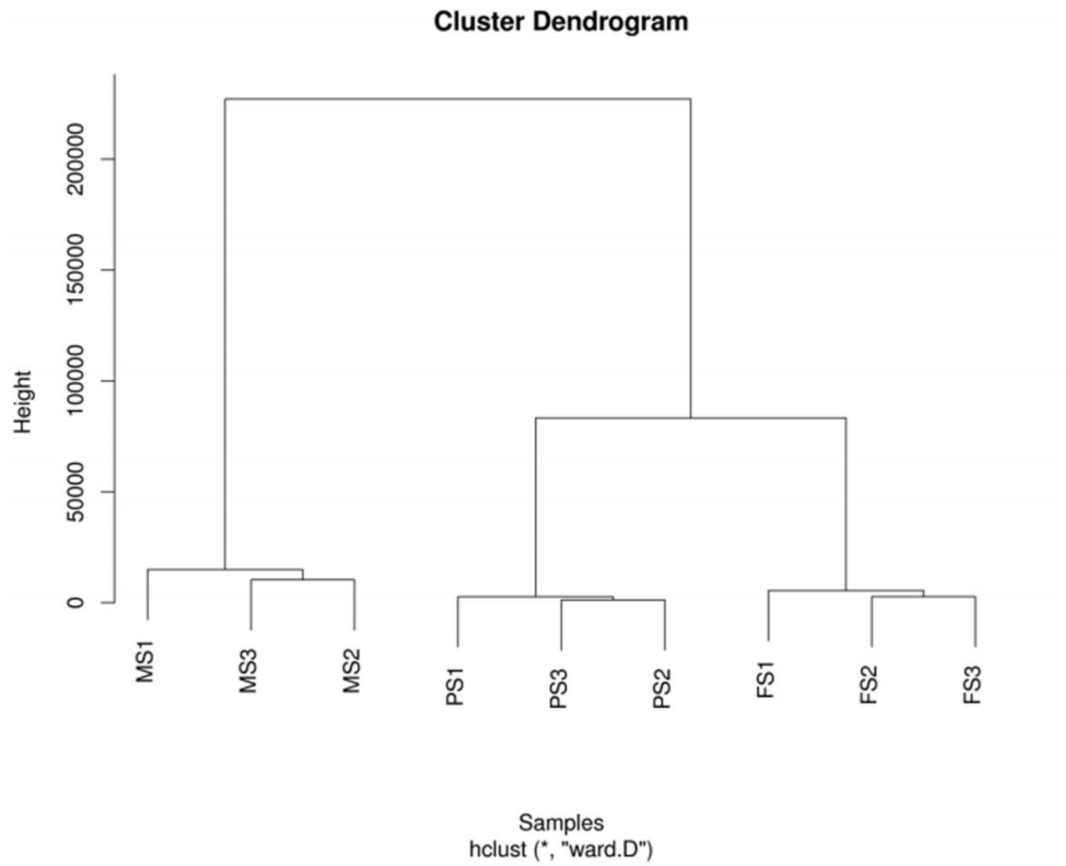

Fig. S1. Cluster dendrogram of nine libraries by hclust method.

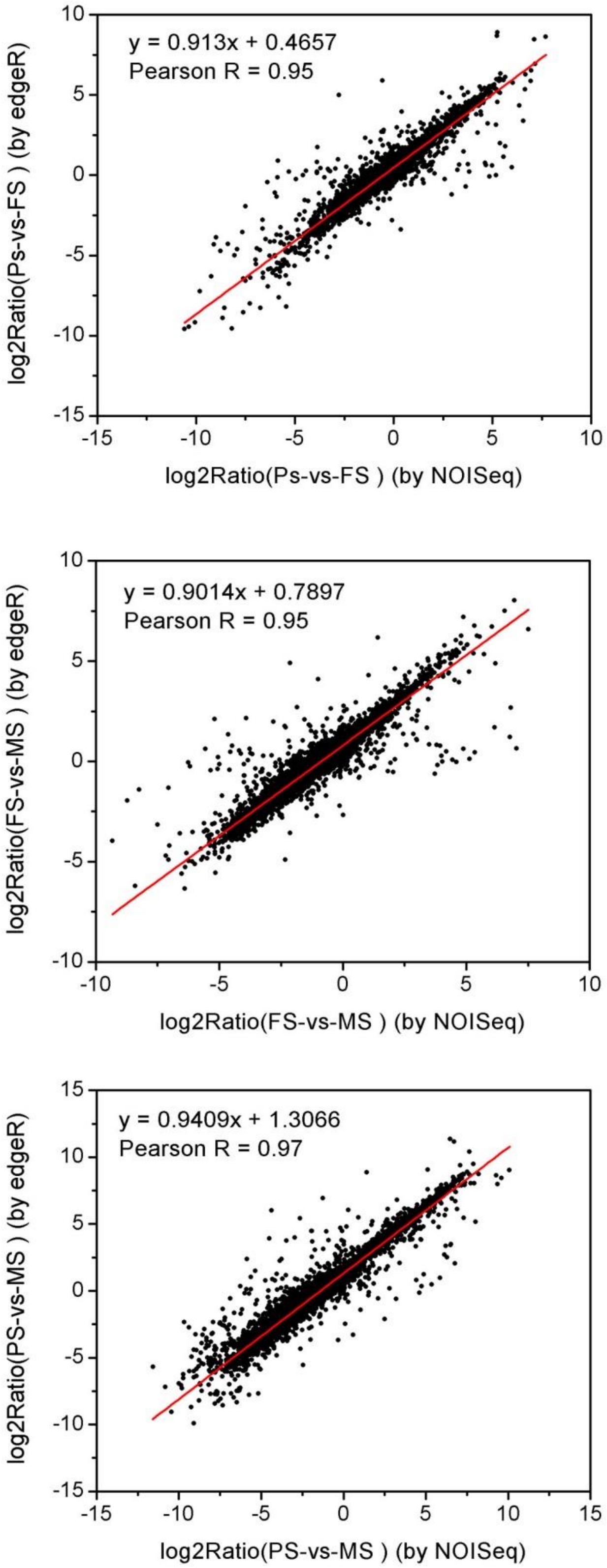

Fig. S2. Comparison of the DEGs obtained by NOISeq and the DEGs obtained by edgeR.

Table S1. Pearson's rank correlation of nine libraries.

Sample	PS1	PS2	PS3	FS1	FS2	FS3	MS1	MS2	MS3
PS1	1	0.989	0.983	0.526	0.486	0.488	0.243	0.218	0.222
PS2	0.989	1	0.996	0.45	0.411	0.413	0.177	0.154	0.158
PS3	0.983	0.996	1	0.431	0.393	0.394	0.158	0.136	0.14
FS1	0.526	0.45	0.431	1	0.991	0.994	0.884	0.881	0.872
FS2	0.486	0.411	0.393	0.991	1	0.998	0.894	0.905	0.889
FS3	0.488	0.413	0.394	0.994	0.998	1	0.89	0.901	0.884
MS1	0.243	0.177	0.158	0.884	0.894	0.89	1	0.983	0.996
MS2	0.218	0.154	0.136	0.881	0.905	0.901	0.983	1	0.993
MS3	0.222	0.158	0.14	0.872	0.889	0.884	0.996	0.993	1

Table S2. List of the stage specifically expressed genes.

GeneID	PS(FPKM)	FS(FPKM)	MS(FPKM)
sample1_00001150-RA	0.05	0.18	4.33
sample1_00001950-RA	0.66	0.59	6.70
sample1_00004426-RA	0.05	0.31	3.21
sample1_00005101-RA	0.07	0.20	2.93
sample1_00005630-RA	0.22	0.17	3.57
sample1_00005766-RA	0.01	1.01	10.84
sample1_00005767-RA	0.03	0.86	6.17
sample1_00006058-RA	0.04	0.02	3.28
sample1_00006530-RA	1.12	1.32	32.02
sample1_00007274-RA	0.15	0.51	24.06
sample1_00009290-RA	0.26	0.96	4.42
sample1_00009424-RA	0.95	3.45	14.54
sample1_00009425-RA	0.37	2.85	16.15
sample1_00009789-RA	2.29	1.45	13.94
sample1_00010413-RA	0.10	0.50	4.71
sample1_00010684-RA	0.14	1.35	89.20
sample1_00014173-RA	2.45	3.84	360.64
sample1_00016081-RA	0.16	0.48	9.93
sample1_00017204-RA	1.74	2.21	11.54
sample1_00017756-RA	0.31	0.34	9.34
sample1_00017973-RA	0.47	1.00	6.32
sample1_00018860-RA	1.32	1.19	14.57
sample1_00019807-RA	0.33	2.08	16.34
sample1_00020491-RA	1.24	0.56	24.42
sample1_00022746-RA	0.80	0.33	4.89
sample1_00026183-RA	0.41	0.84	3.85
sample1_00026465-RA	0.43	1.54	4.03
sample1_00000082-RA	0.24	0.97	5.09
sample1_00000374-RA	8.20	41.94	383.87
sample1_00001123-RA	0.33	2.33	22.83
sample1_00004099-RA	10.47	192.20	1481.39
sample1_00004262-RA	0.55	2.31	15.90
sample1_00009555-RA	0.24	0.56	3.87
sample1_00011242-RA	1.22	9.09	135.80
sample1_00012450-RA	0.45	0.47	7.16
sample1_00018502-RA	0.34	2.42	42.31
sample1_00020744-RA	0.22	0.94	22.81
sample1_00022583-RA	0.24	1.81	27.45
sample1_00025469-RA	0.08	0.42	8.16
sample1_00027191-RA	6.37	19.92	654.76
sample1_00000083-RA	1.18	1.47	6.62

sample1_00000087-RA	0.56	2.03	23.89
sample1_00000628-RA	3.67	2.59	20.14
sample1_00000629-RA	5.69	19.05	415.20
sample1_00000676-RA	3.54	4.44	27.50
sample1_00001460-RA	22.69	107.88	1388.60
sample1_00001911-RA	3.83	9.58	83.52
sample1_00002935-RA	5.85	5.89	72.80
sample1_00003535-RA	24.74	668.88	3612.55
sample1_00004225-RA	18.74	231.29	1507.58
sample1_00005170-RA	22.72	179.62	1628.04
sample1_00005408-RA	5.31	8.95	50.92
sample1_00005419-RA	0.19	1.22	9.79
sample1_00006106-RA	0.06	0.73	2.94
sample1_00007538-RA	0.32	3.23	21.14
sample1_00007672-RA	3.33	51.46	238.66
sample1_00007884-RA	14.10	11.01	133.56
sample1_00008110-RA	12.23	18.42	465.59
sample1_00009090-RA	2.40	10.42	142.49
sample1_00009092-RA	0.17	1.57	7.97
sample1_00009816-RA	0.01	0.82	7.74
sample1_00011427-RA	27.22	199.21	2964.49
sample1_00011449-RA	0.07	1.36	23.70
sample1_00012127-RA	2.09	1.15	8.87
sample1_00012279-RA	0.41	6.07	44.28
sample1_00012319-RA	1.59	3.44	17.15
sample1_00012928-RA	20.22	157.91	1401.02
sample1_00013130-RA	73.34	1578.17	9269.46
sample1_00013685-RA	1.22	0.65	3.99
sample1_00014166-RA	10.26	13.83	198.31
sample1_00014172-RA	78.14	1375.25	8407.58
sample1_00014740-RA	3.60	9.80	48.82
sample1_00015095-RA	0.43	1.15	3.48
sample1_00015096-RA	3.02	64.97	242.04
sample1_00015258-RA	30.02	128.95	663.25
sample1_00015507-RA	4.56	40.70	174.25
sample1_00015832-RA	10.24	111.85	714.56
sample1_00016926-RA	3.84	9.49	61.05
sample1_00017256-RA	2.47	1.16	5.96
sample1_00017522-RA	0.06	0.95	3.57
sample1_00018030-RA	2.34	7.07	47.55
sample1_00018139-RA	2.27	5.02	20.59
sample1_00018181-RA	2.83	8.32	177.86
sample1_00019102-RA	2.13	2.50	26.74
sample1_00019606-RA	0.46	0.61	3.76

sample1_00019961-RA	4.14	10.11	263.21
sample1_00020223-RA	0.01	0.25	4.07
sample1_00020401-RA	3.06	31.56	170.85
sample1_00020501-RA	0.28	0.51	4.76
sample1_00020512-RA	20.83	195.22	1365.07
sample1_00020627-RA	0.06	0.19	2.86
sample1_00020743-RA	0.14	0.12	4.96
sample1_00020745-RA	0.49	1.66	18.50
sample1_00021423-RA	1.91	5.90	42.78
sample1_00021661-RA	0.94	13.96	82.45
sample1_00021775-RA	1.44	4.13	20.50
sample1_00022130-RA	40.59	458.37	2595.35
sample1_00022554-RA	0.47	2.21	24.11
sample1_00022665-RA	10.12	41.48	1022.38
sample1_00023161-RA	49.72	418.40	2451.89
sample1_00023843-RA	0.56	3.72	87.75
sample1_00024808-RA	5.70	30.49	273.21
sample1_00025337-RA	12.11	46.13	284.44
sample1_00025443-RA	0.72	2.27	14.83
sample1_00025444-RA	34.09	339.34	3606.08
sample1_00025879-RA	0.55	0.41	8.40
sample1_00026585-RA	0.09	1.07	10.04
sample1_00026876-RA	22.50	198.39	875.77

Table S4. Biological processes of the DEGs significantly enriched based on GO annotation (FDR≤0.05).

GO_acc	Term	C1_P VALUE	C1_FDR	C2_P VALUE	C2_FDR	C3_P VALUE	C3_FDR	C4_P VALUE	C4_FDR	C5_P VALUE	C5_FDR	C6_P VALUE	C6_FDR	C7_P VALUE	C7_FDR	C8_P VALUE	C8_FDR
GO:0019318	hexose metabolic process	#N/A	#N/A	0.0000015	0.00054	0.12	1	#N/A	#N/A	#N/A	#N/A	#N/A	#N/A	0.98	1	0.97	1
GO:0005996	monosaccharide metabolic process	#N/A	#N/A	0.000004	0.0007	0.15	1	#N/A	#N/A	#N/A	#N/A	#N/A	#N/A	0.97	1	0.97	1
GO:0006006	glucose metabolic process	#N/A	#N/A	0.000017	0.0011	0.052	1	#N/A	#N/A	#N/A	#N/A	#N/A	#N/A	0.98	1	0.99	1
GO:0006066	alcohol metabolic process	#N/A	#N/A	0.000028	0.0011	0.24	1	#N/A	#N/A	#N/A	#N/A	#N/A	#N/A	0.95	1	0.97	1
GO:0006007	glucose catabolic process	#N/A	#N/A	0.000029	0.0011	#N/A	#N/A	#N/A	#N/A	#N/A	#N/A	#N/A	#N/A	0.95	1	#N/A	#N/A
GO:0019320	hexose catabolic process	#N/A	#N/A	0.000029	0.0011	#N/A	#N/A	#N/A	#N/A	#N/A	#N/A	#N/A	#N/A	0.95	1	#N/A	#N/A
GO:0044275	cellular carbohydrate catabolic process	#N/A	#N/A	0.000029	0.0011	#N/A	#N/A	#N/A	#N/A	#N/A	#N/A	#N/A	#N/A	0.95	1	#N/A	#N/A
GO:0046164	alcohol catabolic process	#N/A	#N/A	0.000029	0.0011	#N/A	#N/A	#N/A	#N/A	#N/A	#N/A	#N/A	#N/A	0.95	1	#N/A	#N/A
GO:0046365	monosaccharide catabolic process	#N/A	#N/A	0.000029	0.0011	#N/A	#N/A	#N/A	#N/A	#N/A	#N/A	#N/A	#N/A	0.95	1	#N/A	#N/A
GO:0016052	carbohydrate catabolic process	#N/A	#N/A	0.00008	0.0028	#N/A	#N/A	#N/A	#N/A	#N/A	#N/A	#N/A	#N/A	0.99	1	0.96	1
GO:0044262	cellular carbohydrate metabolic process	#N/A	#N/A	0.00017	0.0055	0.0084	1	#N/A	#N/A	#N/A	#N/A	#N/A	#N/A	0.95	1	0.99	1
GO:0044282	small molecule catabolic process	#N/A	#N/A	0.00035	0.01	#N/A	#N/A	#N/A	#N/A	#N/A	#N/A	#N/A	#N/A	0.93	1	0.97	1

GO:0005975	carbohydrate metabolic process	#N/A	#N/A	0.00038	0.01	0.035	1	#N/A	#N/A	#N/A	#N/A	0.051	1	1	1	0.89	1
GO:0006631	fatty acid metabolic process	#N/A	#N/A	0.0019	0.048	#N/A	#N/A	#N/A	#N/A	#N/A	#N/A	#N/A	#N/A	0.84	1	0.83	1
GO:0032787	monocarboxylic acid metabolic process	#N/A	#N/A	0.002	0.048	#N/A	#N/A	#N/A	#N/A	#N/A	#N/A	#N/A	#N/A	0.8	1	0.76	1
GO:0006950	response to stress	#N/A	#N/A	0.64	1	0.12	1	0.0036	0.092	0.028	0.74	#N/A	#N/A	1	1	0.25	1
GO:0009628	response to abiotic stimulus	#N/A	#N/A	#N/A	#N/A	#N/A	#N/A	#N/A	#N/A	0.00043	0.049	#N/A	#N/A	0.99	1	0.13	1
GO:0010467	gene expression	#N/A	#N/A	1	1	1	1	#N/A	#N/A	0.99	1	#N/A	#N/A	1.2E-12	3.2E-09	1	1
GO:0043170	macromolecule metabolic process	0.95	1	0.98	1	1	1	0.71	1	0.99	1	0.89	1	1.2E-10	0.00000017	1	1
GO:0016043	cellular component organization	#N/A	#N/A	#N/A	#N/A	#N/A	#N/A	#N/A	#N/A	#N/A	#N/A	#N/A	#N/A	0.000000017	0.000016	1	1
GO:0006412	translation	#N/A	#N/A	#N/A	#N/A	#N/A	#N/A	#N/A	#N/A	#N/A	#N/A	#N/A	#N/A	0.00000018	0.00012	1	1
GO:0051276	chromosome organization	#N/A	#N/A	#N/A	#N/A	#N/A	#N/A	#N/A	#N/A	#N/A	#N/A	#N/A	#N/A	0.00000026	0.00015	1	1
GO:0043933	macromolecular complex subunit organization	#N/A	#N/A	#N/A	#N/A	#N/A	#N/A	#N/A	#N/A	#N/A	#N/A	#N/A	#N/A	0.00000037	0.00015	1	1
GO:0006325	chromatin organization	#N/A	#N/A	#N/A	#N/A	#N/A	#N/A	#N/A	#N/A	#N/A	#N/A	#N/A	#N/A	0.00000037	0.00015	1	1
GO:0034621	cellular macromolecular complex subunit organization	#N/A	#N/A	#N/A	#N/A	#N/A	#N/A	#N/A	#N/A	#N/A	#N/A	#N/A	#N/A	0.00000042	0.00015	1	1
GO:0006996	organelle organization	#N/A	#N/A	#N/A	#N/A	#N/A	#N/A	#N/A	#N/A	#N/A	#N/A	#N/A	#N/A	0.0000012	0.00038	0.98	1
GO:0044085	cellular component biogenesis	#N/A	#N/A	#N/A	#N/A	#N/A	#N/A	#N/A	#N/A	#N/A	#N/A	#N/A	#N/A	0.0000026	0.00073	1	1
GO:0043038	amino acid activation	#N/A	#N/A	#N/A	#N/A	#N/A	#N/A	#N/A	#N/A	#N/A	#N/A	#N/A	#N/A	0.0000034	0.00078	#N/A	#N/A

GO:0043039	tRNA aminoacylation	#N/A	#N/A	#N/A	#N/A	#N/A	#N/A	#N/A	#N/A	#N/A	#N/A	#N/A	#N/A	0.0000034	0.00078	#N/A	#N/A
GO:0006418	tRNA aminoacylation for protein translation	#N/A	#N/A	#N/A	#N/A	#N/A	#N/A	#N/A	#N/A	#N/A	#N/A	#N/A	#N/A	0.000005	0.0011	#N/A	#N/A
GO:0044267	cellular protein metabolic process	#N/A	#N/A	0.97	1	1	1	#N/A	#N/A	0.63	1	#N/A	#N/A	0.000018	0.0035	0.98	1
GO:0046483	heterocycle metabolic process	#N/A	#N/A	0.97	1	#N/A	#N/A	#N/A	#N/A	#N/A	#N/A	#N/A	#N/A	0.000024	0.0045	1	1
GO:0033036	macromolecule localization	#N/A	#N/A	0.59	1	#N/A	#N/A	#N/A	#N/A	#N/A	#N/A	#N/A	#N/A	0.000036	0.0062	1	1
GO:0008104	protein localization	#N/A	#N/A	#N/A	#N/A	#N/A	#N/A	#N/A	#N/A	#N/A	#N/A	#N/A	#N/A	0.000039	0.0063	1	1
GO:0006163	purine nucleotide metabolic process	#N/A	#N/A	#N/A	#N/A	#N/A	#N/A	#N/A	#N/A	#N/A	#N/A	#N/A	#N/A	0.000046	0.007	1	1
GO:0019538	protein metabolic process	#N/A	#N/A	0.98	1	0.99	1	0.36	1	0.84	1	#N/A	#N/A	0.000055	0.0078	0.96	1
GO:0034728	nucleosome organization	#N/A	#N/A	#N/A	#N/A	#N/A	#N/A	#N/A	#N/A	#N/A	#N/A	#N/A	#N/A	0.000056	0.0078	0.98	1
GO:0045184	establishment of protein localization	#N/A	#N/A	#N/A	#N/A	#N/A	#N/A	#N/A	#N/A	#N/A	#N/A	#N/A	#N/A	0.000065	0.0082	1	1
GO:0015031	protein transport	#N/A	#N/A	#N/A	#N/A	#N/A	#N/A	#N/A	#N/A	#N/A	#N/A	#N/A	#N/A	0.000065	0.0082	1	1
GO:0006753	nucleoside phosphate metabolic process	#N/A	#N/A	0.87	1	#N/A	#N/A	#N/A	#N/A	#N/A	#N/A	#N/A	#N/A	0.000071	0.0082	1	1
GO:0009117	nucleotide metabolic process	#N/A	#N/A	0.87	1	#N/A	#N/A	#N/A	#N/A	#N/A	#N/A	#N/A	#N/A	0.000071	0.0082	1	1

GO:0055086	nucleobase, nucleoside and nucleotide metabolic process	#N/A	#N/A	0.9	1	#N/A	#N/A	#N/A	#N/A	#N/A	#N/A	#N/A	#N/A	0.000083	0.0092	1	1
GO:0009259	ribonucleotide metabolic process	#N/A	#N/A	#N/A	#N/A	#N/A	#N/A	#N/A	#N/A	#N/A	#N/A	#N/A	#N/A	0.000095	0.01	1	1
GO:0006259	DNA metabolic process	#N/A	#N/A	#N/A	#N/A	#N/A	#N/A	#N/A	#N/A	#N/A	#N/A	#N/A	#N/A	0.0001	0.011	0.98	1
GO:0009165	nucleotide biosynthetic process	#N/A	#N/A	#N/A	#N/A	#N/A	#N/A	#N/A	#N/A	#N/A	#N/A	#N/A	#N/A	0.00014	0.014	#N/A	#N/A
GO:0009150	purine ribonucleotide metabolic process	#N/A	#N/A	#N/A	#N/A	#N/A	#N/A	#N/A	#N/A	#N/A	#N/A	#N/A	#N/A	0.00019	0.018	1	1
GO:0006520	cellular amino acid metabolic process	#N/A	#N/A	0.49	1	#N/A	#N/A	#N/A	#N/A	#N/A	#N/A	#N/A	#N/A	0.0002	0.018	0.99	1
GO:0006508	proteolysis	#N/A	#N/A	#N/A	#N/A	#N/A	#N/A	#N/A	#N/A	#N/A	#N/A	#N/A	#N/A	0.0002	0.018	#N/A	#N/A
GO:0034641	cellular nitrogen compound metabolic process	#N/A	#N/A	0.58	1	#N/A	#N/A	#N/A	#N/A	#N/A	#N/A	#N/A	#N/A	0.00023	0.02	0.98	1
GO:0006164	purine nucleotide biosynthetic process	#N/A	#N/A	#N/A	#N/A	#N/A	#N/A	#N/A	#N/A	#N/A	#N/A	#N/A	#N/A	0.0003	0.024	#N/A	#N/A
GO:0022613	ribonucleoprotein complex biogenesis	#N/A	#N/A	#N/A	#N/A	#N/A	#N/A	#N/A	#N/A	#N/A	#N/A	#N/A	#N/A	0.0003	0.024	0.95	1
GO:0034660	ncRNA metabolic process	#N/A	#N/A	#N/A	#N/A	#N/A	#N/A	#N/A	#N/A	#N/A	#N/A	#N/A	#N/A	0.00042	0.033	0.97	1
GO:0044106	cellular amine metabolic process	#N/A	#N/A	0.57	1	#N/A	#N/A	#N/A	#N/A	#N/A	#N/A	#N/A	#N/A	0.00053	0.04	0.99	1
GO:0006754	ATP biosynthetic process	#N/A	#N/A	#N/A	#N/A	#N/A	#N/A	#N/A	#N/A	#N/A	#N/A	#N/A	#N/A	0.00061	0.045	#N/A	#N/A
GO:0046034	ATP metabolic process	#N/A	#N/A	#N/A	#N/A	#N/A	#N/A	#N/A	#N/A	#N/A	#N/A	#N/A	#N/A	0.00061	0.045	#N/A	#N/A
GO:0009141	nucleoside triphosphate metabolic process	#N/A	#N/A	#N/A	#N/A	#N/A	#N/A	#N/A	#N/A	#N/A	#N/A	#N/A	#N/A	0.00067	0.048	1	1
GO:0006350	transcription	#N/A	#N/A	#N/A	#N/A	0.43	1	#N/A	#N/A	#N/A	#N/A	#N/A	#N/A	1	1	5.5E-09	0.0000022
GO:0006351	transcription, DNA-dependent	#N/A	#N/A	#N/A	#N/A	0.43	1	#N/A	#N/A	#N/A	#N/A	#N/A	#N/A	1	1	5.5E-09	0.0000022
GO:0032774	RNA biosynthetic process	#N/A	#N/A	#N/A	#N/A	0.43	1	#N/A	#N/A	#N/A	#N/A	#N/A	#N/A	1	1	5.5E-09	0.0000022

Table S8. List of primers for qRT-PCR analysis.

**ID**	**Annotation**	**Product Length**	**Forward Primer**	Reverse Primer
sample1_00021677-RA	13S globulin	265	ACGACAACGCCATAACCAGT	ACCTCCCTTATCCTTTTCCCTC
sample1_00013128-RA	13S globulin	262	AGGTTGAGGTCTTCCGACCA	CAAGCGATCCCGTTTCTTCG
sample1_00013130-RA	13S globulin	262	AGGTTGAGGTCTTCCGACCA	CAAGCGATCCCGTTTCTTCG
sample1_00019024-RA	7S globulin-like	72	CAGTGCTTGGGGCTAGTTGA	ATCCTCCAACTGCTTGGCTC
sample1_00017374-RA	7S globulin-like	229	TACAACTTCCTTCCGCCACC	CGGTGCTGATCTTGGTTCCT
sample1_00013278-RA	C4H	83	ACGAGAGGAAGAAGCTCGG	TGTGCCTCCAAGATGTGGTC
sample1_00012486-RA	CHI	72	TGCTGTGATTGAAAACGGGC	GGCGAAACGCCATTCTTACC
sample1_00002939-RA	CHS	131	TTGGGGTGTCTTGTTTGGCT	TGAAAATGTAAGGGCGCCGA
sample1_00002940-RA	CHS	108	TGGGGTGTCTTGTTTGGCTT	TGTAAGGGCGCCTAGGGATA
sample1_00006854-RA	CHS	151	AGAAGGCAAGAAGACGACCG	GCTCGCAAACTGACGAAAACT
sample1_00021115-RA	CHS	179	GGAAACATGTCGAGTGCGTG	AAGACTTAGTTGGCCACGGG
sample1_00016770-RA	CHS	277	AGGCGGAGAAGATGAAGGC	TGTGGAGAGACACCATCAGGA
sample1_00006550-RA	F35H	282	TGGTGTCTTGTGCATTGGCT	CAGGGTTATCCTCACGCTCA
sample1_00003908-RA	F3H	72	ATGCAACCGTTTACCCGCTA	CAGCAAAGGTGATTGGTGCC
sample1_00012969-RA	F3'H	277	GGTCTACGGCCCTCTTATGC	CCCTACCTCCTCCTCACGAA
sample1_00021254-RA	F3H	102	AGCCGTTTGTCAATTGCGAC	GATGGGCTCCTCCAAGATCG
sample1_00009801-RA	DFR	268	AGCTGACCGATGAAGGGTTC	AACGAAGCGAAAAGCACCAA
sample1_00013849-RA	FLS	85	CACTCTCAAGGTCGGTGGAC	GACCCTTGGAACGTCGTGAT
sample1_00013850-RA	FLS	168	CCGGAGAATAGCAAGGCACT	GACCGCCTGAACCTTCTTGA
sample1_00005844-RA	LAR	207	GTCGAGGGACCAAACGAAGT	CATGGCTGGTATGGGCAGAA
sample1_00017445-RA	LAR	164	AGTCGAGATGACTGTTGCCG	TCTTGGAAGGGGAAATGGGC
sample1_00013708-RA	LAR	108	GAAGATGAAAGTCTCCGCCT	GTCTGCCGGAATCCAAGGTT
sample1_00011838-RA	UGT	97	GGATGCTGCCAACGCTAATG	ACTGCAAGCTTGGTAGGAAGT

sample1_00009713-RA	AAO3	130	GCCGTGTAAAGTTGGTCGTT	CACATTCCTTCCTCGTCTATCTG
sample1_00009748-RA	CYP707A2	142	TTTCGCTGCTCACGACACTAC	TGTGAGACCTCGTCCAGTAGAAT
sample1_00009778-RA	CYP707A3	207	TTGCAGCACAAGACACCACA	CAGGAATCACATACCATCCGTTA
sample1_00009427-RA	CYP707A3	179	CACAACACTTCCCTGACCCA	TGTGATGCTCCACCTGTATTTT
sample1_00026417-RA	PP2CA	206	GGAGCCAGAGGTGACCATAA	ACATTGCCGCATCAGAGC
sample1_00021697-RA	ABI3	180	AACGGTCTGATGGTTCTTGG	CATTGTCATCCCAACATTCGT
sample1_00015232-RA	LEC1	100	CAACGCAAGACGATAACAGC	GATAGCGATGGAGATAAAGGGT
sample1_00002821-RA	ETR1	130	TTGTTGAAGTAGTTGCCGACC	TCTGCTTCCCTCTTAGCCATA
sample1_00002766-RA	IPT8	135	AAACCATCCGCTCTTAGTCG	AACGAAACATCCACCCAGAG
sample1_00003907-RA	JMT	164	TACAAGACCCGAATTGAATCAG	AAGCAACCACCTACACTCCCT
sample1_00026062-RA	GA3OX3	180	TTATGGAGGCGTTCCTGTTG	ATAACCTTCTCCGCCACCA
FtActin	-	192	GAGTTATGAGCTTCCTGATG	CCGCCACTCAACACAATGTT
